# Random forest analysis reveals taxa predictive of Prunus replant disease in peach root microbiomes

**DOI:** 10.1371/journal.pone.0275587

**Published:** 2022-10-13

**Authors:** Abdur R. Khan, Wisnu A. Wicaksono, Natalia J. Ott, Amisha T. Poret-Peterson, Greg T. Browne

**Affiliations:** 1 Department of Plant Pathology, University of California, Davis, California, United States of America; 2 USDA-ARS Crops Pathology and Genetics Research Unit, Davis, California, United States of America; Savitribai Phule Pune University, INDIA

## Abstract

Successive plantings of *Prunus* species produce suboptimal growth and yield in many California soils due to a poorly understood soilborne disease complex, Prunus replant disease (PRD). We explored the hypothesis that PRD is mediated by microbial taxa in roots of Nemaguard peach, a rootstock for almond and other stone fruits. In a greenhouse bioassay, portions of 10 replant soils were treated with fumigation or pasteurization or left untreated as a control before being planted with peach seedlings. Ten weeks after planting, seedlings were considered PRD-affected if their top fresh weights in the control were significantly reduced, compared to the weights in pasteurization and fumigation treatments; plants with equivalent top weights in all treatments were considered to be non-affected. The roots were washed from the soil, frozen, extracted for total DNA, and used for metabarcoding of rRNA gene amplicons from bacteria, fungi, and oomycetes. High-throughput amplicon sequencing revealed that root microbial community shifts resulted from preplant treatments, and specific taxa were associated with PRD induction among controls. Random forest (RF) analysis discriminated effectively between PRD-affected and non-affected root communities. Among the 30 RF top-ranked amplicon sequence variant (ASV) predictors, 26 were bacteria, two were oomycetes, and two were fungi. Among them, only *Streptomyces scabiei*, *Steroidobacter denitrificans*, *Streptomyces bobili*, and *Pythium mamillatum* had root abundances ≥5% that were either associated positively (former two ASVs) or negatively (latter two) with PRD. Thus, our findings were consistent with microbial mediation of PRD in roots and suggested taxa that may be involved in the mediation.

## Introduction

Successive orchard plantings of almond and other stone fruits (*Prunus* species) often exhibit poor growth and productivity due to one or more replant problems, some well-understood and others poorly defined. In the former category are several species of phytopathogenic nematodes (PPN), which can feed and reproduce on or in roots of *Prunus* and cause long-term orchard decline [1–3]. PPN tend to be relatively non-specific in their capacity for damage among fruit and nut species; for example, *Criconemella xenoplax* populations that affect grape also affect subsequently planted *Prunus* spp. PPN do not occur universally among replant soils, and monitoring of their soil and/or root populations can be used to partially assess the need for soil remediation. On the other hand, in the category of poorly defined replant problems, many soils that have hosted *Prunus* species, even in the absence of PPN, induce Prunus replant disease (PRD), a relatively host-specific problem that affects, for example, *Prunus* planted after *Prunus* but not *Prunus* after apple, citrus, or grape [4,5] (Browne, *unpublished*). Trees affected by PRD exhibit stunting, reduced root length density, and, in severe cases, failure to establish [6]. Preplant soil fumigation with chloropicrin-containing fumigants, anaerobic soil disinfestation (ASD) with rice bran, and soil pasteurization all can prevent PRD, suggesting biological mediation of the disease [6,7]. Abiotic replant problems, such as nutrient deficiencies or toxicities, pH imbalance, and soil compaction also have been recognized, and they may interact with biotic replant problems [3,4].

Due to its practical significance, PRD etiology has long been researched, but its apparent complexities and variations among soils have eluded convergence on single or core causes. As early as 1941, a “peach replant problem” not associated with plant parasitic nematodes or other known pathogens was reported in California. Peach planted after peach was affected, but not peach after apple. Addition of peach root bark or alcohol extracts from the roots induced root disease and poor growth of peach seedlings in potted sand. Subsequent reports suggested that biologically-mediated hydrogen cyanide production from peach root residues contributed to the peach replant problem [8,9]. Indeed, many studies over the last 40 years have provided evidence that peach root system residues can contribute to PRD and that microbial community members can mediate aspects of the process [10–12]. In contrast, Hine [13] contended that while peach root residues and cyanogenesis were short-lived in soil, the capacity for a replant problem in old peach orchard soil was persistent. Also, soils from non-affected and affected replant sites had an equivalent capacity for cyanide production from peach root residues [14]. Hine and Wensley [14,15] independently provided evidence for roles of soil borne fungi and/or oomycetes in replant problems of peach. Bent et al. associated high abundances of *Sellaphora* spp. and *Pythium* spp. with growth suppression of peach in a replant soil [16], while Yang et al. associated root abundances of *Phytopythium vexans* and *Ceratocystis fimbriata* with peach replant suppression [17].

Continued application of high-throughput sequencing technologies and bioinformatics offer promise for resolving causes of recalcitrant replant problems. For example, recent research using such methods with apple replant disease (ARD), which shares some complexities with PRD, discovered associations among several *Streptomyces* species and the apple malady [18]. In the current report, we focus on root communities associated with PRD induction; HTS and bioinformatics approaches including random forest (RF) classification modelling were used to identify features of the root microbial communities that were predictive of PRD induction or absence.

## Materials and methods

### Soil collection and treatment

Root microbial communities examined in this report were sampled from Nemaguard peach seedlings at the conclusion of a greenhouse bioassay involving 10 replant soils. The soils were collected in spring 2015 from 10 differently managed areas of land distributed across northern, central, and southern portions of California’s Central Valley. Physicochemical and microbial community features of these soils, as well 15 additional soils, were reported previously, including rRNA gene-amplicon-based characterizations of bacterial, fungal and oomycete communities and physical-extraction-based characterizations of resident nematode populations [19]. At the time of soil collection, capacity of the soils to induce PRD generally was not known. A catalogued list of the soils and their GPS locations is provided (S1 Table). All of the soil collection locations were either privately owned or on University of California (UC) research property; permission for sampling was obtained in all cases. Eight of the soils had recent crop histories of almond or peach on peach rootstock (soils numbers 1, 3, 6, 10, 13–15 and 23; S1 Table), and two were from grape vineyards (soils 11 and 12). Trees had been removed several months before collection of soils 13–15, whereas crops were still standing in the other soils. Soils 13, 14, and 15 were removed from the same field, but, before collection, had received separate soil treatments *in situ* (control, fumigation, and anaerobic soil disinfestation, respectively) as described previously [20]. Each of the 10 soils was collected at three or four random sampling locations within a represented field, and, at each sampling location, an 8-cm-diameter hand auger was used to collect soil at two or three points at depths of 10 to 61 cm. The soil from each sampling location was pooled, mixed, and stored at 10 to 22°C until use in the greenhouse bioassay.

### Greenhouse bioassay

In preparation for the bioassay, each soil was mixed with autoclaved sand 2:1 (soil:sand v:v) to facilitate adequate soil water drainage. The sand had been autoclaved at 121°C for 45 m on three successive days and then allowed to stand for 2 or more days before the mixing. The mixtures were apportioned to three preplant treatments: a non-treated control, preplant fumigation with chloropicrin, and preplant pasteurization with steam. The portions allocated to the control treatment were stored at 15 to 22°C in vented polyethylene bags. Portions allocated to fumigation were also placed in polyethylene bags and then nested in 19-liter buckets (one bag and bucket per soil) that had been lined with a totally impermeable film (TIF) (Raven, Sioux Falls, SD, USA); the soil in each bucket was injected with 3 ml of chloropicrin (TriCal, Inc.; Hollister, CA, USA), quickly sealed in the TIF, and then, 1 week after treatment, allowed to vent thoroughly. Soil mixture portions allocated to pasteurization were injected with steam through multiple nozzles at the bottom of 18-liter metal containers to maintain soil temperature at ≥80°C for 30 min, then allowed to cool.

For the bioassay, the treatment-soil-source combinations were distributed to 0.9-L pots in a randomized complete block design. There were six blocks, each containing one pair of pots (i.e., paired pots were subsamples) per treatment-soil source combination. All of the pots were planted with single recently sprouted peach seedlings (*Prunus persica* × *P*. *davidiana* ‘Nemaguard’, a common rootstock for stone fruits and nuts (Sierra Gold Nursery, Inc., Yuba City, CA, USA). The plants were grown in a greenhouse with air temperatures of 16 to 30°C and potted soil temperatures of 17 to 29°C. All plants were watered daily as needed with a modified Hoagland’s solution [21].

Ten weeks after planting, the seedlings were assessed for growth by determining the plant top fresh weights. For each plant, all stem, shoot, and leaf materials were collected and weighed after cutting from the main stem at 1 cm above the soil surface. The weights were subjected to analysis of variance (ANOVA) using PROC MIXED of SAS Version 9.4 (SAS, Cary NC, USA). The model statement for PROC MIXED specified plant top weight as a function of preplant soil treatment, soil source, and interaction of preplant soil treatment × soil source; block was specified as a random variable. Plant top fresh weight means were separated according to 95% confidence intervals. Plants with average top fresh weights significantly lower in the control treatment, compared to fumigated and pasteurized treatments for the same soils, were considered to be affected by PRD. Plants with statistically equivalent fresh weights resulting from control, fumigated, and pasteurized treatments of a soil were considered not to be affected by PRD in the soil. The ratio of plant top fresh weight in control soil divided by the average top fresh weight in fumigated and pasteurized portions of the soil was calculated and is henceforth referred to as the “control proportion of top weight”; the smaller the proportion, the greater the severity of PRD.

### Root sampling and DNA extraction

At the conclusion of the greenhouse bioassay, the root systems were gently but thoroughly washed free from soil, frozen immediately on dry ice, and stored at -80°C. DNA extractions were completed from roots in three randomly selected blocks. The extractions occurred from roots of one randomly selected plant per preplant soil treatment. Each extraction used 2 to 10 g of fine roots (≤ 1 to 2 mm diameter), kept frozen while grinding into powder using an MM 200 Mixer Mill homogenizer (Retsch, Newton, PA, USA). Total DNA was extracted from 100-mg subsamples of the ground tissues using the MoBio PowerPlant Pro kit and further purified with MoBio PowerClean Pro kit (Mo Bio Laboratories, Inc., Carlsbad, CA, USA). The quality and yield of the extracted DNA was determined using the Nanodrop 2000 UV-Vis spectrophotometer (Thermo Fisher Scientific Inc., Waltham, MA, USA) and Qubit DNA ds HS assay system (Thermo Fisher Scientific Inc., Waltham, MA, USA), respectively, following the manufacturer’s instructions.

### Amplicons and Illumina sequencing

To manage potential biases of individual primer sets, two sets of primers were used to amplify rRNA gene regions from each microbial domain and kingdom of interest, including bacteria, fungi, and oomycetes. Primer sets included, for the 16S rRNA gene in bacteria: 515F/806R [22] targeting the V4 region, and 799F/1193R [23] targeting V5-V7 regions; in fungi: ITS1f/ITS2 and fITS7/ITS4 targeting ITS1 and ITS2 regions, respectively [24]; and, in oomycetes: ITS1oo/ITS7 and ITS3oo/ITS4, targeting the ITS1 and ITS2 regions, respectively [25] (S2 Table).

A two-step PCR approach was used with dual indexing (DI) (S2 Table) as previously described [26,27]. The first reaction used 25 μL mixtures containing 1x buffer, 0.2mM deoxynucleotide triphosphates (dNTPs), 2.5 mM MgCl_2_, 2 μM tagged forward primer, 2 μM tagged reverse primer (Integrated DNA Technologies, Inc, USA), 3% dimethyl sulfoxide (DMSO) (Sigma Aldrich, USA), 0.5 U KAPA2G HotStart DNA Polymerase (KAPA Biosystems, Woburn, MA, USA) and 20–30 ng template DNA. A total of 15 cycles was used in the first PCR for both bacterial primer sets following previously described reaction parameters [22,23]. The first PCR for fungi and oomycetes consisted of 20 cycles following published parameters [24,25,28], with the modification of increasing the first denaturation temperature from 94 to 95°C. The second PCR was performed using the reagents as described for the first PCR, except that 1 μL of product from the first PCR mixture was used as template, and barcoded primers, targeting the “tags” on primers in the first PCR, were used at a concentration of 2 μM. The second PCR cycling parameters included an initial denaturation of 1 min at 95°C, followed by 10 cycles of: 30 sec denaturation at 95°C, 30 sec of annealing at 60°C, 1 min of extension at 68°C, and 5 min final extension at 68°C.

Five μL from each final PCR product was examined via gel electrophoresis. Band brightness of each PCR product was compared, relative to 1kb Plus ladder (Thermo Fisher Scientific, Waltham, MA, USA) with ImageJ [29], and then pooled to achieve similar concentrations prior to PCR purification, as described previously [30]. Agencourt AMPure magnetic beads 0.8x were used to purify the libraries (Beckman Coulter, Sacramento, CA, USA). Amplicon sequencing was performed using Illumina MiSeq (v2 reaction kit) (2 × 300 bp paired-end) by the DNA Technologies Core Facility at University of California Davis, USA. Sequences were deposited into the NCBI Sequence Read Archive (SRA, BioProject Number PRJNA491510).

### Sequence processing

The raw sequences were processed using QIIME2 version 2019.4.0 [31]. Raw paired-end FASTQ files were demultiplexed with demux plugin, and primer sequences were removed using cutadapt plugin [32]. Most paired sequence reads generated from oomycete primers ITS3oo and ITS4 (ITS2 libraries) could not be merged due to poor sequencing quality for read 2; therefore, we used only read 1 generated from oomycete ITS2 sequencing. All other sequence sets were merged successfully. Sequences were quality filtered, checked to remove chimeras, trimmed, denoised, and merged using the DADA2 algorithm [33]. Each bacterial amplicon sequence variant (ASV) resulting from DADA2 analysis was taxonomically assigned using the Ribosomal Database Project (RDP) naive Bayesian rRNA classifier [34] with Silva v132 [35]. Prior to the analysis, non-bacterial sequences from chloroplasts and mitochondria were excluded from the libraries. Fungal ASVs were assigned using the Basic Local Alignment Search Tool (BLAST) with UNITE + INSD (v6_sh_97) [36]; ASVs not classified to a fungal phylum were then compared with ITS fungal sequences in the NCBI nr database using the BLAST [37]. Oomycete ASVs were assigned using the BLAST with the NCBI nr database. Reads belonging to non-fungal or non-oomycete taxa, in the respective datasets, were removed before further analysis. Further data analyses were conducted using packages developed for R software version 3.4.2 [38] in the RStudio development environment [39], as well as PRIMER version 7 software [40] and Paleontological statistics software (Past) version 4.01 [41].

### Normalization and general analysis, all-treatment sample sets

Rarefication was first conducted among amplicons from all soil treatment sample sets (i.e., control, pasteurization, and fumigation) to assess effects of the treatments on root microbial community structure (S3 Table). To compensate for differences in sequencing depth, the bacterial V4 and V5-V7 libraries were rarefied to 1,365 and 793 reads, and six samples were removed from each of these libraries due to low read numbers (85 to 671) (S3 Table). The fungal ITS1 and ITS2 libraries were rarefied to 1,589 and 918 reads, respectively, and four samples were removed from ITS2 libraries due to low read numbers (305–712) (S3 Table). Among the oomycete ITS1 and ITS2 sample libraries, 47 and 64%, respectively, had read counts of less than 550, and those with low read numbers were predominately from the fumigated and pasteurized soil treatments. For this reason, examinations of the oomycete root communities were restricted to libraries from the non-treated control soil treatment (see below, under “normalization and general analysis of control treatment sample sets”).

The rarified bacterial and fungal libraries were analyzed using the ‘phyloseq’ package [42] to determine richness (number of observed ASVs, Chao1), Shannon diversity, and relative abundances of taxa. Analysis of variance (ANOVA) was conducted using vegan R packages [43] through RStudio [39] to test for effects of preplant soil treatment on microbial richness and diversity estimates; Tukey’s honest significant difference (HSD) was used for mean separation at *P* = 0.05. Bar plots were generated at class level for bacteria and fungi, and at genus level for oomycetes using PRIMER version 7 software [40].

Next, compositional effects of the soil treatments on bacterial and fungal communities associated with roots were assessed using permutational analysis of variance (PERMANOVA) and analysis of similarity (ANOSIM). In preparation, bacterial and fungal ASVs that occurred in fewer than two samples with counts ≥ 1 were excluded, and cumulative sum scaling (CSS) normalization was then completed in the metagenomeseq package in R [44,45]. The CSS-normalized ASV counts were transformed (square root for bacterial ASVs, natural log [x+1] for fungal ASVs) and used to generate BC-Dissimilarity matrices in PRIMER 7. The BC-Dissimilarity matrices were subjected to PERMANOVA and ANOSIM in PRIMER7; PERMANOVA+ was used with 9,999 permutations [40]. Also, in PRIMER7, the RELATE test (9,999 permutations) was used to compare the BC-dissimilarity matrices resulting from the complementary PCR primer sets, and non-metric multidimensional scaling (NMDS) was used to visualize microbial community differences based on BC-dissimilarity.

### Normalization and general analysis, control treatment sample sets

Rarefication was then conducted for amplicons from the control soil treatment sample sets to examine, among them, root microbial community aspects that may differ between PRD-inducing and non-inducing soils (S3 Table). Using R, V4 and V5-V7 amplicon libraries were rarefied to 1,526 and 978 reads, respectively; fungal ITS1 and ITS2 libraries were rarefied to 3,165 and 1,524 reads, respectively; and oomycete ITS1 and ITS2 libraries were rarefied to 564 and 644 reads, respectively. Rarefication resulted in exclusion of one fungal ITS2 library and one oomycete ITS2 library.

Diversity indices were determined and compared among rarified amplicon libraries from control sample sets as was described for libraries from the all-soil-treatment sample sets, except that the former diversity index means were compared using pairwise t-tests implemented in PAST version 4.01 [41]. Also, in preparation for PERMANOVA, ASVs that occurred in fewer than two samples with counts ≥ 1, were excluded, followed by CSS normalization. BC-Dissimilarity matrices generated using square-root-transformed (bacteria) and natural log [x+1] transformed (fungi and oomycetes) ASV counts. The BC-Dissimilarities were subjected to PERMANOVA to determine whether the PRD-inducing capacity of the soils (i.e., inducing vs. non-inducing, as determined by the greenhouse bioassay) significantly impacted composition of the peach root microbial communities. PERMANOVA was implemented using 9,999 permutations in PRIMER 7. NMDS, also implemented in PRIMER 7, was used to visualize the BC-dissimilarities.

### Random forest analysis, control treatment library sets

We used random forest (RF) classification [46] to identify root community features that discriminated between PRD-affected and non-affected samples. “RandomForests” package version 4.6–14 [47] was used in R. PRD status of the communities (i.e., PRD-affected or non-affected, as determined in the greenhouse bioassay) was used as the qualitative response variable. The script for small data sets (n = 30) was used as recommended [48]. ASV counts from V4, ITS1 fungal, and ITS2 oomycete amplicons were pooled and filtered within each sample to remove ASVs occurring less than five times. The remaining counts, which were for 853 ASVs, were CSS-normalized. The normalized counts were transformed in PRIMER 7 (square-root for V4, natural log [x+1] for ITS1 fungal and ITS2 oomycete) in preparation for RF analysis. In the RF classification model, a total of 801 trees in parameter “ntree” was used. Packages including “rfUtilities” and “caret” in R were used to test model significance and to get leave-one-out cross-validation accuracies, respectively. The function “Importance” was used to assess the predictive value of each ASV for RF classification [46]. Pearson’s correlation coefficients were calculated using Paleontological statistics software (Past) version 4.01 [41] to relate relative abundances of top-ranked predictive ASVs to control top fresh weight proportions (our measure of PRD severity).

## Results

### Greenhouse bioassay

**Fig 1:**
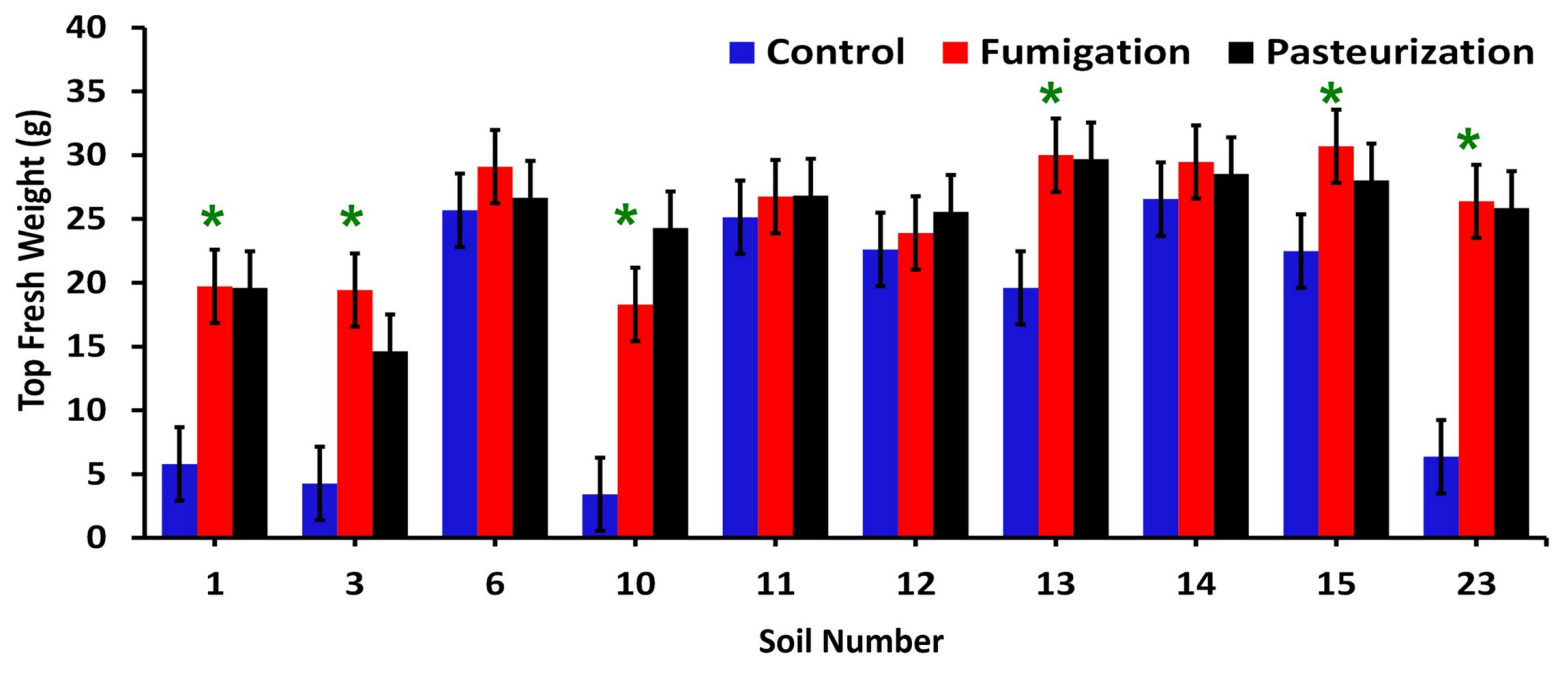
Final top fresh biomass of Nemaguard peach seedlings as a function of soil source and preplant soil treatment in greenhouse bioassay. The weights were affected by significant soil source × soil treatment interaction (*P*<0.0001). Asterisks indicate soils in which mean top fresh weights from fumigated and pasteurized treatments exceeded the weights of the control treatment, according to 95% confidence intervals indicated by error bars.

In the bioassay, there was a significant interactive effect of soil source and preplant soil treatment on plant top fresh weight (*P*<0.0001) (Fig 1). Based on 95% confidence intervals for the interactive means, preplant soil fumigation and pasteurization had equivalent effects on the plant top fresh weights; both treatments resulted in equivalent, significant increases in top, root, and total fresh weights in six of the 10 soils (nos. 1, 3, 10, 13, 15, and 23), and neither treatment affected plant weights, compared to the control, in the other four soils (nos. 6, 11, 12, and 14). (Fig 1).

### Effects of preplant soil treatments on bacterial root communities

In the V4 amplicon libraries representing samples from all preplant soil treatments, bacterial root community richness as revealed by observed ASVs and Chao1 estimates varied significantly according to preplant soil treatment (*P*<0.0001, based on ANOVA; Table 1). Numbers of observed ASVs and Chao1 estimates were roughly 1.5 to 1.7 times greater in the non-treated control treatment than in the fumigated and pasteurized treatments (Table 1). In contrast, neither Shannon diversity in the V4 libraries nor any of the richness or diversity indices in the V5-V7 amplicon libraries was affected significantly by preplant soil treatment (*P* = 0.55 and 0.20 to 0.98, respectively; ANOVA) (Table 1).

**Table 1:** Richness and diversity of bacterial and fungal community rRNA gene amplicon libraries from roots as a function of primer set and preplant soil treatment^a^.

Target region (primer set)	Preplant soil treatment	Observed ASVs	Chao1 estimate	Shannon diversity
**Bacterial V4 (515F-806R)**	**Control**	106.1±8.81 a	123.1±12.28 a	3.2±0.14 a
	**Fumigation**	67.2±4.02 b	69.8±4.62 b	3.29±0.08 a
	**Pasteurization**	71.7±3.95 b	76.3±4.91 b	3.36±0.05 a
**Bacterial V5-V7 (799F-1193R)**	**Control**	56.1±4.24 a	59.8±4.93 a	3.1±0.1 a
	**Fumigation**	49±3.34 a	53.3±4.47 a	3.1±0.08 a
	**Pasteurization**	47.2±3.44 a	51.8±5.05 a	3.1±0.06 a
**Fungal ITS1 (ITS1f-ITS2)**	**Control**	35±3.42 a	40.2±4.05 a	1.9±0.17 a
	**Fumigation**	19.6±1.36 b	20.9±1.44 b	1.4±0.09 b
	**Pasteurization**	14.5±1.33 b	14.9±1.35 b	1.2±0.09 b
**Fungal ITS2** **(fITS7-ITS4)**	**Control**	42.4±2.6 a	49.7±2.94 a	2.2±0.14 a
	**Fumigation**	22.2±1.52 b	24.1±1.69 b	1.6±0.1 b
	**Pasteurization**	17±1.22 b	18.4±1.41 b	1.4±0.1 b

^a^ Mean±SE calculated for each treatment. Values in the same column and within a primer set are significantly different (*P*<0.05) if they have different letters, according to Tukey HSD test for multiple comparisons.

**Fig 2:**
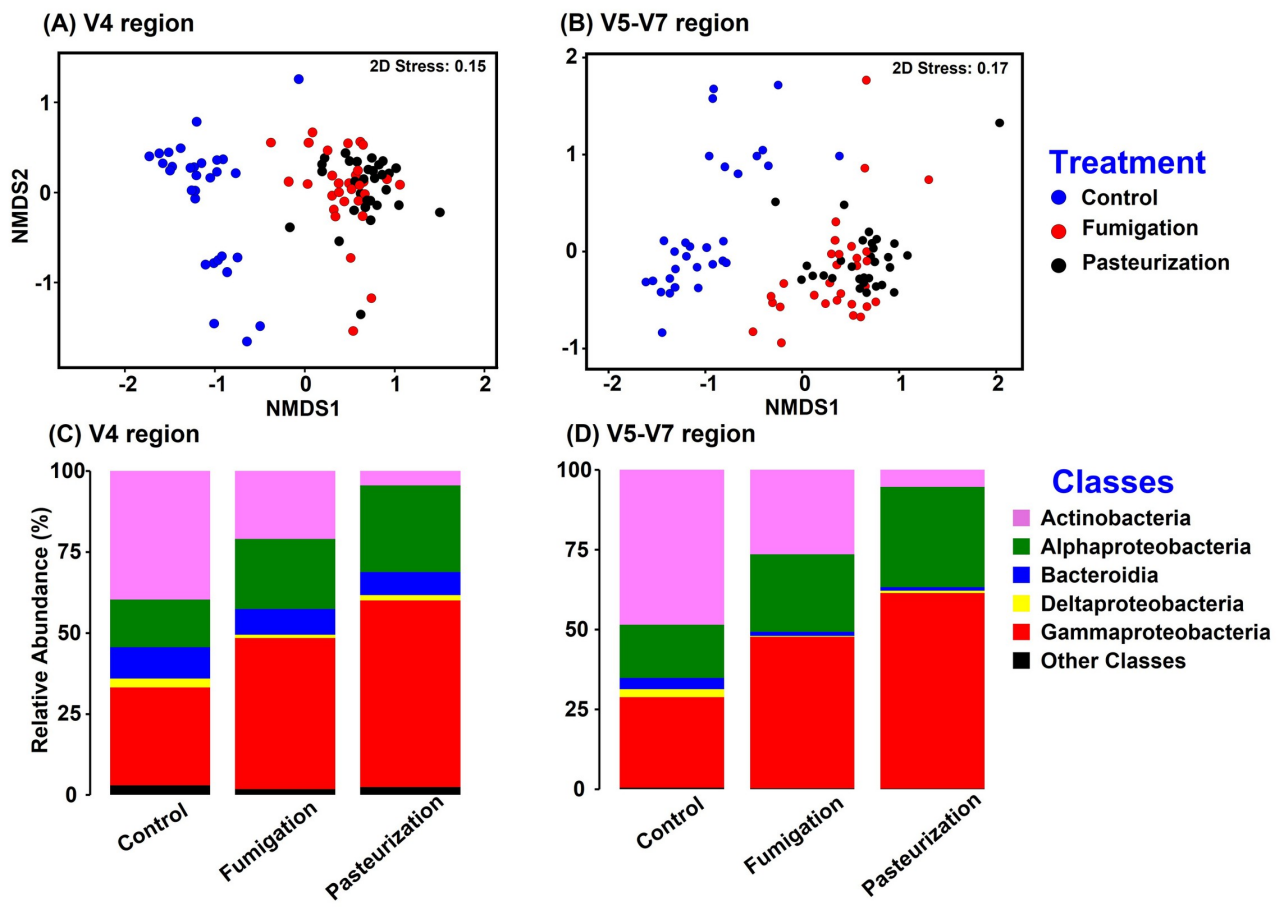
Response of bacterial communities in peach roots to preplant soil treatments among samples from the greenhouse bioassay. **A** and **B**, non-metric multidimensional scaling (NMDS) ordinations of the communities based on Bray-Curtis dissimilarities of ASV communities revealed by V4 (primers 515-806R) and V5-V7 (primers 799F-1193R), respectively; and **C** and **D**, combined class-level summary of ASV relative abundances based on V4 and V5-V7 amplicons, respectively. For NMDS, Bray-Curtis dissimilarity matrices were generated after cumulative sum scaling (CSS) normalization and square-root-transformation. The class level abundances were calculated after rarefication.

Global PERMANOVA and ANOSIM of BC-Dissimilarities at the ASV level indicated significant effects of preplant soil treatments on bacterial root communities (PERMANOVA: pseudo-F = 22.86, *P* = 0.0001 for V4 region; and pseudo-F = 20.85, *P* = 0.0001 for V5-V7 region; ANOSIM: R = 0.60, *P* = 0.001 for V4 region; and R = 0.54, *P* = 0.001 for V5-V7 region). Furthermore, for both primer sets, bacterial root community composition differences were significant between all pairs of preplant soil treatments (pairwise PERMANOVA, *P* = 0.0001; ANOSIM, *P* = 0.001) (Table 2). NMDS ordination of the BC-dissimilarities generally clustered bacterial root communities from samples of untreated soil separately from those from samples of fumigated and pasteurized soil (Fig 2A and 2B). BC-dissimilarities based on V4 vs. V5-V7 amplicons corresponded closely, based on the RELATE test (*Rho* = 0.902, *P* = 0.0001). Altogether, ASVs of class Actinobacteria generally had higher relative abundance in roots from untreated soils (39% in V4 amplicons, 37% in V5-V7 amplicons) than in roots from fumigated soils (21% in V4, 26.5% in V5-V7) and pasteurized soils (4.4% in V4, 5.4% in V5-V7) (*P* = 0.001 to 0.06; Fig 2C and 2D). On the other hand, Gammaproteobacteria were more abundant in roots from fumigated soil (46% in V4, 43% in V5-V7) and pasteurized soils (57% in V4 and 55% in V5-V7) than in roots from non-treated control soils (30% in V4; 37% in V5-V7, respectively) (*P* = 0.0003 to 0.001; Fig 2C and 2D).

**Table 2:** Effects of preplant soil treatments on bacterial and fungal root communities as revealed by pairwise PERMANOVA (permutational analysis of variance) and ANOSIM (analysis of similarities) of Bray-Curtis dissimilarities.

Target region (primer set)	Pairwise comparison	PERMANOVA		ANOSIM	
		t	*P*	R	*P*
**Bacterial V4 (515F-806R)**	**Control vs Fumigation**	4.05	0.0001	0.762	0.001
	**Control Vs Pasteurization**	4.39	0.0001	0.834	0.001
	**Fumigation vs Pasteurization**	2.03	0.0001	0.251	0.001
**Bacterial V5-V7 (799 F-1193R)**	**Control vs Fumigation**	3.74	0.0001	0.653	0.001
	**Control Vs Pasteurization**	4.22	0.0001	0.741	0.001
	**Fumigation vs Pasteurization**	2.05	0.0001	0.234	0.001
**Fungal ITS1 (ITS1f-ITS2)**	**Control vs Fumigation**	3.20	0.0001	0.595	0.001
	**Control Vs Pasteurization**	4.00	0.0001	0.809	0.001
	**Fumigation vs Pasteurization**	1.97	0.0001	0.182	0.001
**Fungal ITS2 (fITS7-ITS4)**	**Control vs Fumigation**	3.14	0.0001	0.403	0.001
	**Control Vs Pasteurization**	3.58	0.0001	0.525	0.001
	**Fumigation vs Pasteurization**	2.23	0.0001	0.151	0.008

### Effects of preplant soil treatments on fungal root communities

In fungal ITS and ITS2 amplicon libraries representing samples from all preplant soil treatments, richness and diversity indices were significantly greater in roots from non-treated control soils than in roots from fumigated and pasteurized soils, based on ANOVA comparisons at *P*<0.05 (Table 1). Among these libraries, roots from non-treated soils had roughly double the observed ASVs and Chao1 estimates, compared to roots from fumigated and pasteurized soil treatments (Table 1). Similarly, for both amplicon sets, Shannon diversity estimates were approximately 1.4 to 1.6 times greater in roots from non-treated soils than in roots from fumigated and pasteurized soils.

**Fig 3:**
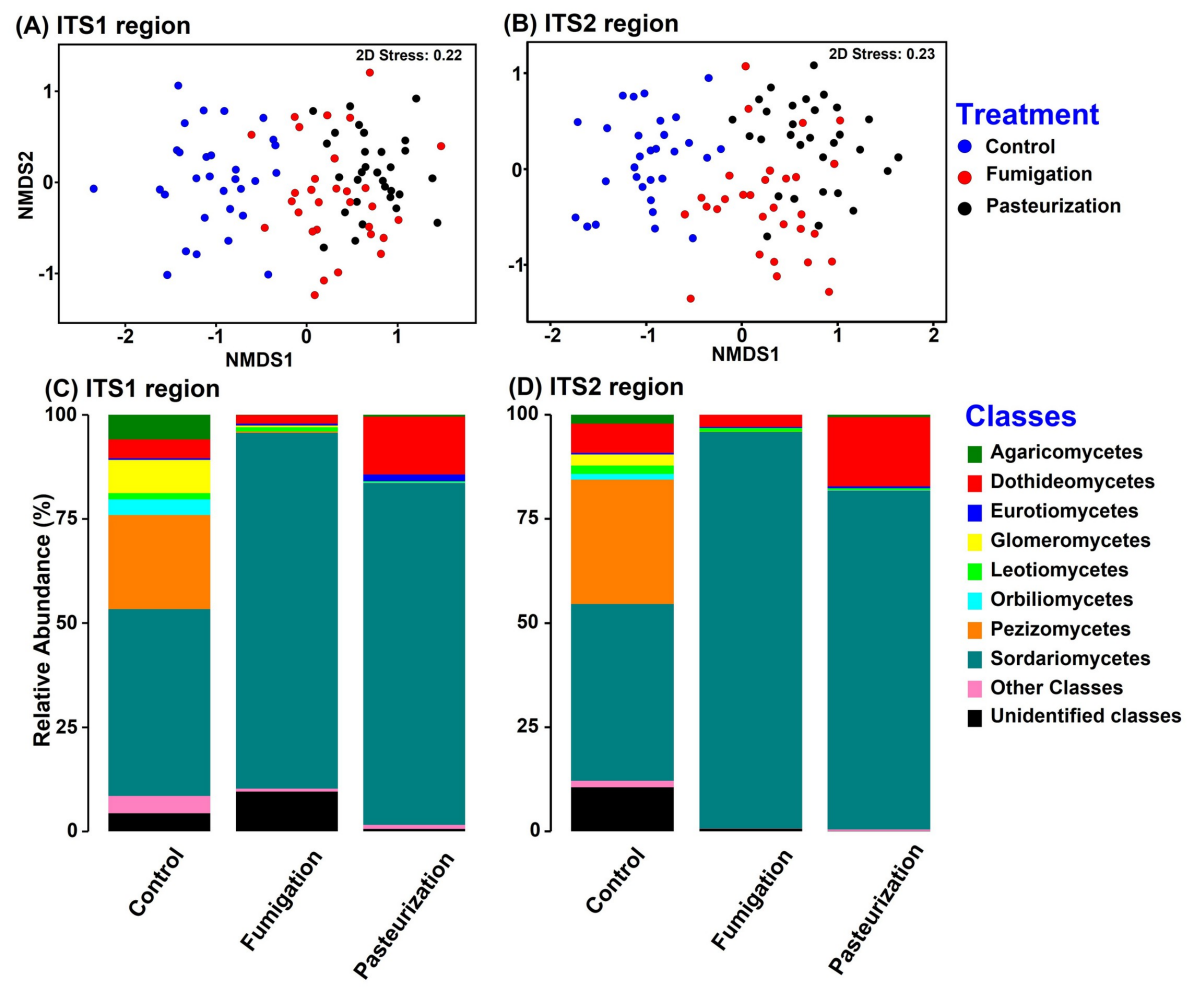
Response of fungal communities in peach roots to preplant soil treatments in 10 replant soils used for the greenhouse bioassay. **A** and **B**, non-metric multidimensional scaling (NMDS) ordinations of the root communities based on BC-dissimilarities among ITS1 (primers ITS1f / ITS2) and ITS2 (primers fITS7 / ITS4) amplicons, respectively; **C** and **D**, class-level summarization of ASVs from ITS1 and ITS2 amplicons, respectively. For NMDS, Bray-Curtis dissimilarity matrices were generated after cumulative sum scaling (CSS) normalization and square-root-transformation. The class level abundances were calculated after rarefication.

Global PERMANOVA and ANOSIM of BC-Dissimilarities at ASV level indicated significant compositional effects of the preplant soil treatments on fungal root communities (PERMANOVA: pseudo-F = 14.89, *P* = 0.0001 for ITS1 region; pseudo-F = 13.4, *P* = 0.0001 for ITS2 region, and ANOSIM: R = 0.517; *P* = 0.001 for ITS1 and R = 0.351 *P* = 0.001 for ITS2). Furthermore, among both sets of amplicons, pairwise PERMANOVA and ANOSIM tests indicated there were significant fungal root community composition differences between each pair of soil treatments (*P* = 0.0001 to 0.008, Table 2). There was close correspondence between ITS1 and ITS2 ASV community structure, based on the RELATE test (*Rho* = 0.744, *P* = 0.0001). NMDS of the fungal BC-dissimilarities at ASV level generally ordinated non-treated control root community samples separately from those of fumigation and pasteurization samples, but the ordinations had high stress (2-D stress 0.22 to 0.23; Fig 3A and 3B). Altogether, ASVs classified as Sordariomycetes had higher relative abundance in roots from fumigated soil (85% ITS1, 95%, ITS2) and pasteurized soil (82% ITS1, 81% ITS2) than in roots from control soil (45% ITS1, 43% ITS2) (*P* = 0.001 for all treatment-control comparisons; Fig 3C and 3D). Conversely, Pezizomycete ASVs had a total higher relative abundance in roots from non-treated soil (22% ITS1, 30%, ITS2) than in roots from fumigated soils (0.32% ITS1, 0.008% ITS2) (*P* = 0.02) and pasteurized soils (0.013% ITS1, 0.008% ITS2) (*P*<0.0001 to 0.02, depending on comparison; Fig 3C and 3D).

### Bacterial root community shifts associated with PRD induction, non-treated soil

**Fig 4:**
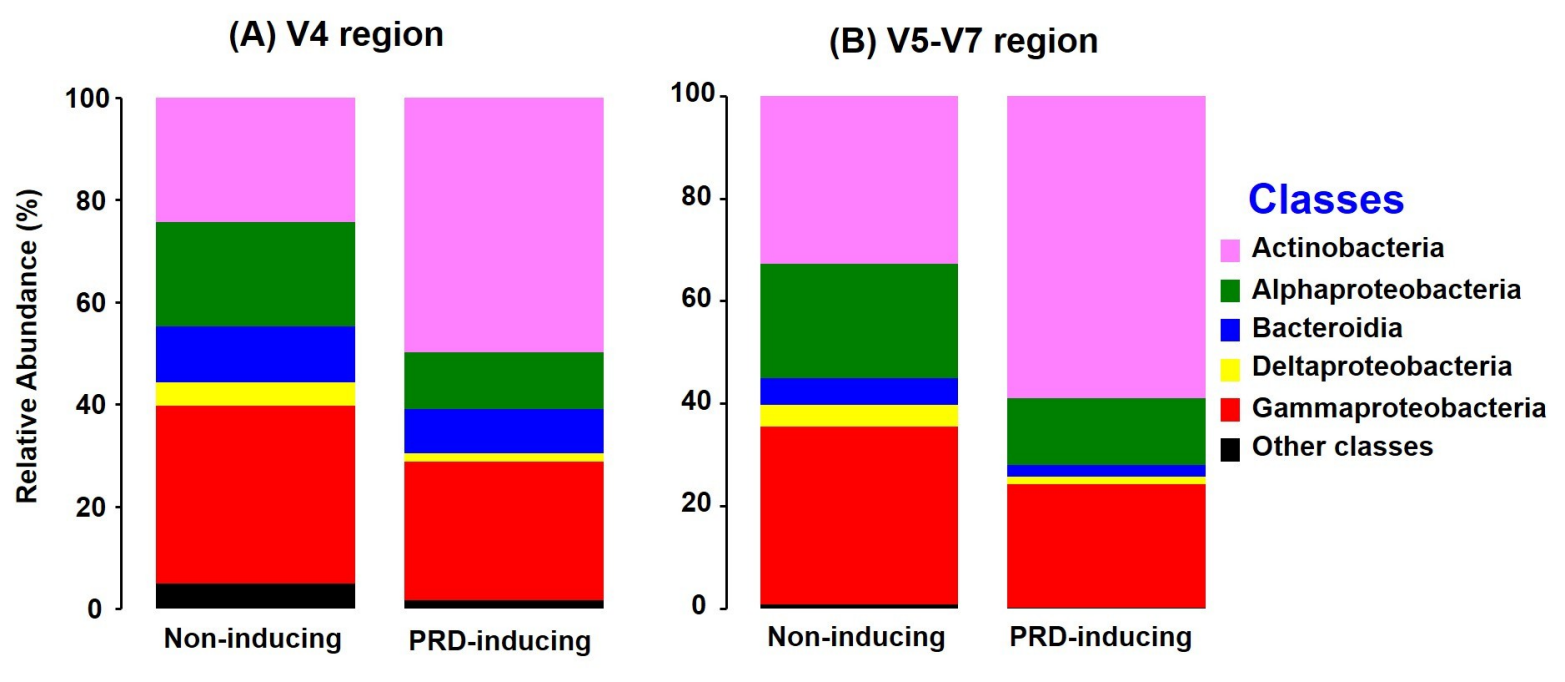
Composition of bacterial communities in peach roots from the six soils that were PRD-inducing and four that were non-inducing in the greenhouse bioassay. **A and B**, class-level relative abundances of ASVs resolved from amplicons of V4 (primers 515F-806R) and V5-V7 (primers 799F-1199R) regions of the rRNA gene, respectively. The soil portions (controls) were those left untreated before the bioassay. ASV abundances were calculated after rarefication.

In bacterial root communities from non-treated soil samples, observed ASVs and Chao 1 estimates were similar between PRD-inducing soils (i.e., soil nos. 1, 3, 10, 13, 15, and 23) and non-inducing soils (nos. 6, 11, 12, and 14), both with V4 and V5-V7 amplicons (*P* = 0.62 to 0.82) (Table 3). However, with both primer sets, Shannon diversity was about 1.25 times greater in roots from non-inducing soils than in roots from PRD-inducing soils (Table 3; *P* = 0.001 to 0.002). Bacterial community composition differed significantly between roots from PRD-inducing and non-inducing soils, as indicated by PERMANOVA (pseudo-F = 6.53 and *P* = 0.0001 for V4 region, pseudo-F = 6.16 and *P* = 0.001 for V5-V7 region) and ANOSIM (R = 0.54 and *P* = 0.001 for V4, R = 0.51 and *P* = 0.001 for V5-V7) (Table 4, S1A and S1B Fig). Altogether, ASVs in class Actinobacteria had higher abundance in roots from PRD-inducing soils (49.4% V4, 57.6% V5-V7) than in roots from non-inducing soils (24.2% V4, 31.7% V5V7) (*P*<0.0001 to 0.0002; Fig 4A and 4B). Conversely, Alphaproteobacteria were less prevalent in roots from PRD-inducing soils (11% V4, 12.8% V5-V7) than in roots from non-inducing soils (20% V4, 23% V5-V7) (*P* = 0.0007 to 0.006).

**Table 3:** Diversity indices among bacterial, fungal, and oomycete root microbial communities as a function of soil PRD-inducing capacity^a^.

Target region (primer set)	Induction capacity	Observed ASVs	Chao1 estimate	Shannon diversity
Bacterial V4 (515F-806R)	PRD-inducing	104.5±12.8	127.4±18.6	2.9±0.2*
	Non-inducing	108.6±11.3	116.8±13.5	3.7±0.2
V5-V7 region (799F-1193R)	PRD-inducing	60.3±6.0	65.2±6.9	2.8±0.1*
	Non-inducing	68.4±6.9	70.2±7.6	3.6±0.1
Fungal ITS1 (ITS1f-ITS2)	PRD-inducing	34.7±2.6	38.5±3.1	1.6±0.2*
	Non-inducing	46±8.7	48.4±9.6	2.4±0.2
Fungal ITS2 (fITS7-ITS4)	PRD-inducing	34.6±2.6*	42.4±2.8*	2.1±0.2
	Non-inducing	46.8±4.1	60.8±4.9	2.3±0.2
Oomycete ITS1 (ITS1oo-ITS7)	PRD-inducing	12.2±1.7	12.5±1.9	1.5±0.1
	Non-inducing	12.5±1.3	13.2±1.6	1.6±0.1
Oomycete ITS2 (ITS3oo-ITS4)	PRD-inducing	8.9±0.7	9.1±0.7	1.3±0.1
	Non-inducing	10.8±0.8	11.6±1.2	1.4±0.2

^a^ Mean±SE calculated on a per sample basis. Values in the same column and primer set with asterisk “*” symbol are significantly different (*P*<0.05), according to pairwise t test.

**Table 4:** Effects of the PRD-inducing capacity of non-treated soil on bacterial, fungal, and oomycete root community composition, as revealed by PERMANOVA (permutational analysis of variance) and ANOSIM (analysis of similarity) of Bray-Curtis dissimilarities^a^.

Target region (and primer set)	PERMANOVA		ANOSIM	
	Pseudo-F	*P*	R	*P*
**Bacterial V4 (515F-806R)**	6.53	0.001	0.54	0.001
**Bacterial V5-V7 (799F-1193R)**	6.16	0.001	0.51	0.001
**Fungal ITS1 (ITS1f-ITS2)**	3.31	0.001	0.37	0.001
**Fungal ITS2 (fITS7-ITS4)**	2.93	0.001	0.26	0.008
**Oomycete ITS1 (ITS1oo-ITS7)**	1.93	0.049	0.08	0.085
**Oomycete ITS2 (ITS3oo-ITS4)**	3.83	0.002	0.18	0.011

^a^PERMANOVA and ANOSIM were conducted to test the significance of PRD-inducing capacity of non-treated soils, as determined by the greenhouse bioassay, on root microbial community structures. The analyses were conducted after CSS normalization and transformation of ASV counts; square-root transformation was used for bacteria, and natural log [x+1] transformation was used for fungi and oomycetes.

### Fungal community shifts associated with PRD-induction, non-treated soil

**Fig 5:**
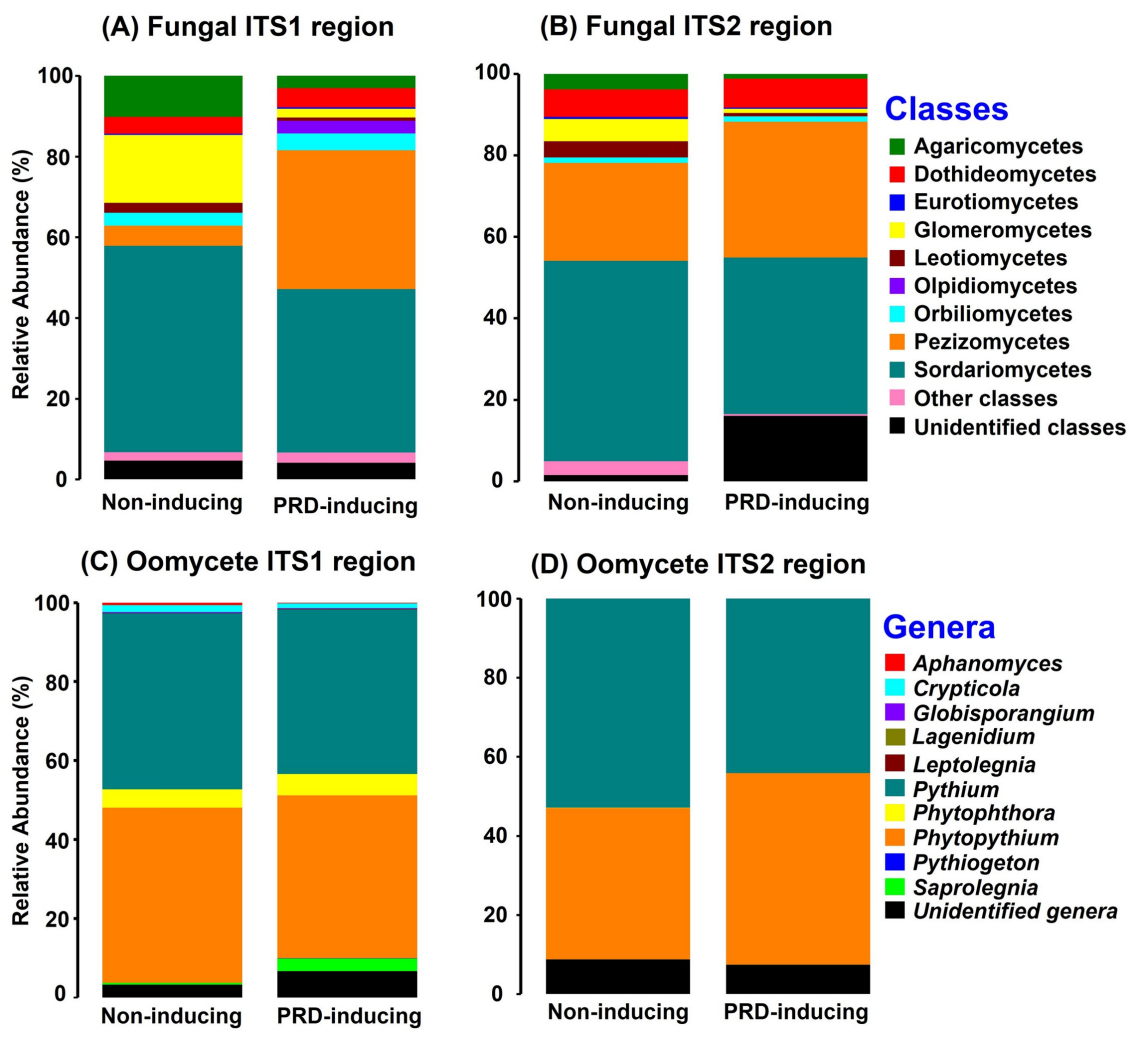
Composition of fungal and oomycete communities in peach roots from the six soils that were PRD-inducing and four that were non-inducing in the greenhouse bioassay. **A** and **B**, class-level relative abundances of fungal ASVs resolved from amplicons of ITS1 (primers ITS1f / ITS2) and ITS2 (primers fITS7 / ITS4) regions of the rRNA gene, respectively; and **C** and **D**, genus-level relative abundances of oomycete ASVs resolved from amplicons of ITS1 (primers ITS1oo-ITS7) and ITS2 (primers ITS3oo-ITS4) regions, respectively. The abundances were calculated after rarefication.

Fungal ITS1 amplicons from roots of non-treated soil samples resolved similar numbers of ASVs and Chao 1 estimates, regardless of the PRD-inducing capacity of the soil (*P* = 0.16 and 0.26, respectively), but there was 1.5 times greater Shannon diversity in the root amplicons from non-inducing soils, compared the root amplicons from PRD-inducing soils (*P* = 0.027; Table 3). In contrast, fungal ITS2 amplicons resolved more ASVs and greater Chao1 estimates in roots from non-inducing soils, compared to in roots from PRD-inducing soils (*P* = 0.01 and 0.002, respectively), but the Shannon diversity was not affected by PRD-inducing status of the soil (*P* = 0.48) (Table 3). PERMANOVA and ANOSIM of ASV-level BC-dissimilarities indicated, both with ITS1 and ITS2 amplicons, significant compositional differences in fungal root communities from PRD-inducing vs. non-inducing soils (PERMANOVA: pseudo-F = 3.31, *P* = 0.001 for ITS1 and pseudo-F = 2.93, *P* = 0.001 for ITS2; ANOSIM: R = 0.37, *P* = 0.003 for ITS1, and R = 0.26, *P* = 0.008 for ITS2) (Table 4 and S1C and S1D Fig). Among the ITS1 amplicons, ASVs in class Pezizomycetes were significantly more abundant in PRD-affected roots (34.1%) than in non-affected roots (5%) (*P* = 0.03), but the difference was not significant among ITS2 amplicons (*P* = 0.51) (Fig 5A and 5B). Glomeromycetes were significantly more abundant in non-affected roots (16% ITS1, 5% ITS2) than in PRD-affected roots (2% ITS1, 1% ITS2) (*P* = 0.004 to 0.005; Fig 5A and 5B).

### Oomycete community shifts associated with PRD-induction, non-treated soils

Oomycete community diversity indices did not differ significantly between root samples from PRD-inducing and non-inducing soils for either ITS1 or ITS2 amplicons (*P* = 0.08 to 0.65; Table 3). Among ITS1 amplicons, PERMANOVA and ANOSIM of BC-dissimilarities revealed a relatively small impact of the PRD-inducing capacity of the soils on oomycete communities in the roots (PERMANOVA pseudo-F = 1.93, *P* = 0.049; ANOSIM R = 0.084, *P* = 0.083) (Table 4 and S1E Fig). However, among the ITS2 amplicons, there was a stronger effect of PRD-inducing capacity on oomycete root communities (PERMANOVA pseudo-F = 3.83, *P* = 0.002; ANOSIM R = 0.177, *P* = 0.011) (Table 4 and S1F Fig). At genus level, *Pythium* and *Phytopythium* dominated the root-associated oomycete communities, both in PRD-inducing and non-inducing soils (Fig 5C and 5D).

### Random forest modeling of PRD-associated root community features

**Fig 6:**
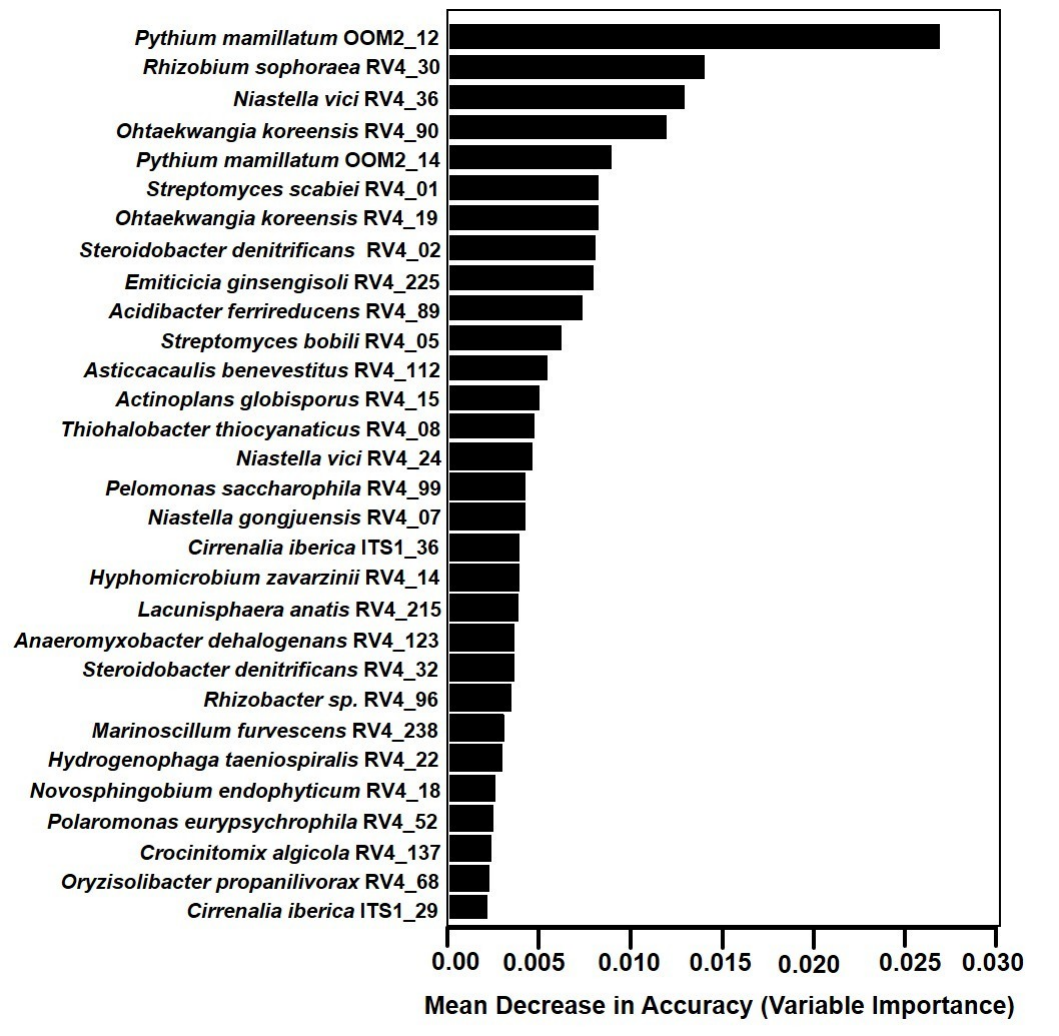
Top-30 ASVs selected as variables in the random forest (RF) classification model, plotted in order of importance. High values of the mean decrease in accuracy indicate more important variables in the RF classification. The taxa names are based on NCBI Blast hit, followed by the assigned ASV numbers.

**Fig 7:**
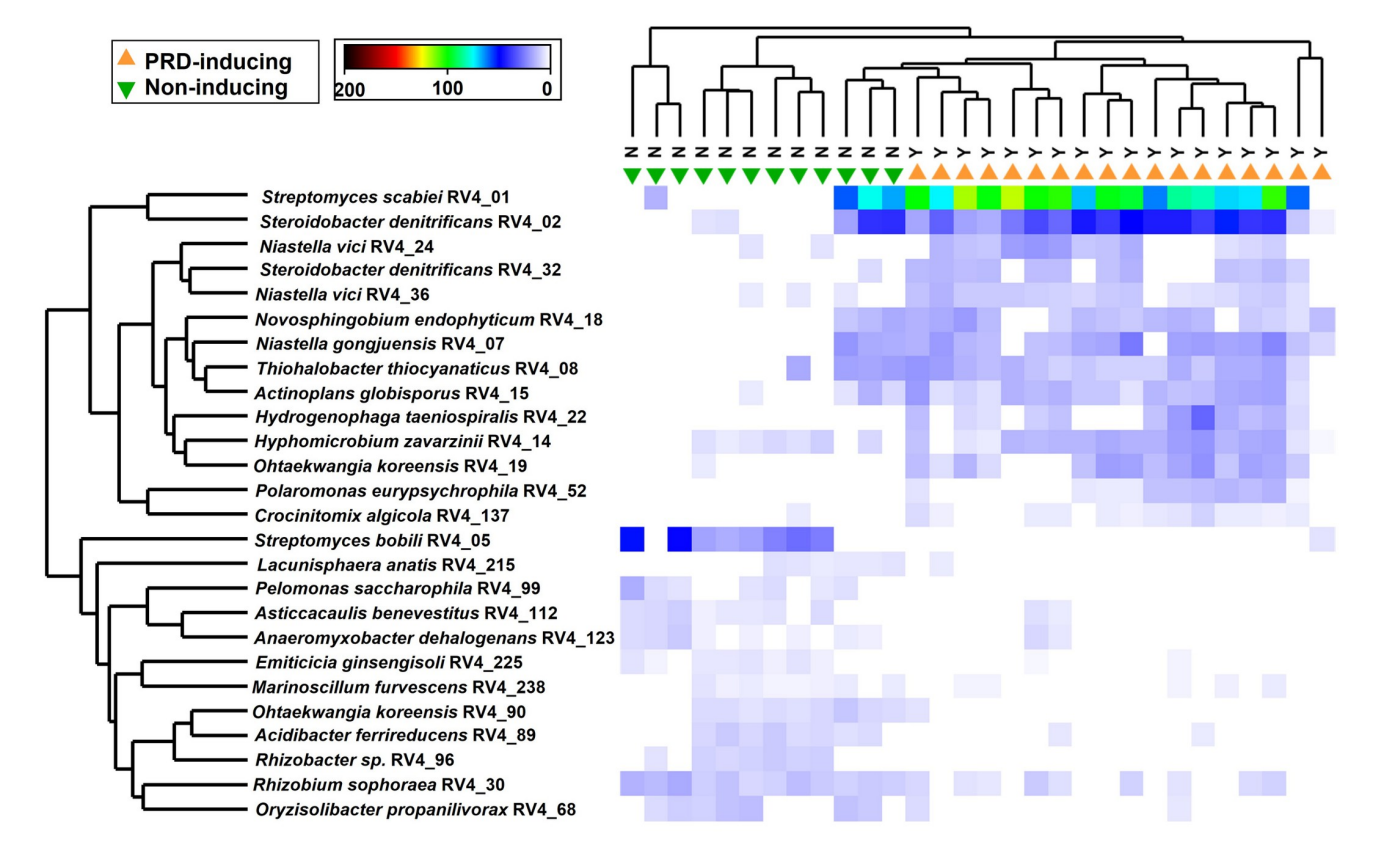
Heatmap and hierarchical clustering of the top-26 bacterial ASVs selected as important variables in the random forest classification model. The heatmap was coded according to square-root transformed abundances (depicted by color intensity) as a function of ASV (rows) and sample (columns). The 30 samples were from non-treated portions of 10 replant soils, with three replicate samples per soil. Hierarchical clustering was based on Bray-Curtis dissimilarities among ASV abundances (among-sample at top and among-ASVs at left). The abundances were CSS-normalized and square-root transformed before generation of Bray-Curtis dissimilarity matrices. ASV labels include: NCBI Blast hit, coding for tissue and primer set (e.g., “RV4” indicated root amplicon amplified by the V4 primers, “OOM2” indicated root oomycetes amplicon amplified by the ITS2 primers), and ASV number.

Based on features of bacterial, fungal, and oomycete ASV abundances in peach root communities from non-treated soils in the greenhouse bioassay, the RF model generally classified samples correctly as PRD-affected or non-affected, yielding an out-of-bag (OOB) error rate of 6.65% for the entire model. The performance of the RF classification model was tested by “leave-one-out” cross-validation, which revealed a classification accuracy of 90% (kappa value of 0.78). When ASVs were ranked according to their mean decrease in prediction value in the classification model, the top 30 predictors included 26 bacterial ASVs, two fungal ASVs, and two oomycete ASVs (Fig 6). Of these, only *Streptomyces scabiei* RV4_01, *Steroidobacter denitrificans* RV4_02, and *Streptomyces bobili* RV4_05 had relative abundances in their respective amplicon sets above 5% in either the PRD-affected or non-affected root systems (Table 5). The two “top-predictive” fungi (*Cirrenalia iberica* ITS1_29 and *C*. *iberica* ITS1_36) had higher abundances in PRD-affected samples, while the two “top-predictive” oomycetes (*Pythium mamillatum* OOM2_12 and *P*. *mamillatum* OOM2_14) had greater mean abundances in non-affected root systems (Table 5). Among the top 26 predictive bacteria, the *Str*. *Scabiei* RV4_01 and *S*. *denitrificans* RV4_02 stood out as generally having relatively high abundances in association with PRD (Fig 7) and significant negative correlations with control proportions of top fresh weight (Table 5), while *Str*. *bobili* RV4_05 stood out with relatively high abundances in non-affected roots and significant positive correlations with control proportions of top fresh weight (Fig 7, Table 5). There were several additional predictive bacterial ASVs with low abundances (≤ 1%) that correlated negatively (r = -0.67 to -0.36, *P*≤ 0.05) or positively (0.36 to 0.70; *P*≤ 0.05) with control proportions of top fresh weight (Table 5, S2 and S3 Figs). Among the bacterial ASVs that were among top-30 RF predictors and exhibited negative correlations with control proportion of top weight, there was a strong tendency for abundances to be lower or absent from the fumigated or pasteurized treatments, compared to their abundances in the control treatment; only *Novosphingobium eodophyticum* RV4_18, whose abundances correlated negatively with control proportions of top weight in non-treated soil, had greater abundances in the former two treatments than in the control (S4 Fig). Among the top-30 bacteria with positive associations to control proportion of top weight, abundances tended to be greater in pasteurized and/or fumigated treatments, except for *Rhizobium sophorae* RV4_30, which had greater abundance in the control (S5 Fig).

**Table 5:** Identities, relative abundances, and relationships to control proportions of top weight among top-30 RF classification model of PRD predictors^a^.

RF-rank	ASV label	NCBI BLAST match, accession number and percent identity (%)	Relative abundance (%)		Correlation with top weight proportion	
			Non-inducing	PRD-inducing	r	*P*
**1**	**OOM2_12**	*Pythium mamillatum*, FJ415930, (100)	5.47	0.000	0.47	0.013
**2**	**RV4_30**	*Rhizobium sophoraea*, MT645963, (100)	1.541	0.145	0.70	0.000
**3**	**RV4_36**	*Niastella vici*, NR_148859, (96.8)	0.059	0.475	-0.39	0.032
**4**	**RV4_90**	*Ohtaekwangia koreensis*, NR_117435, (93.7)	0.537	0.010	0.51	0.004
**5**	**OOM2_14**	*Pythium mamillatum*, FJ415929.1, (100)	1.80	0.000	0.35	0.075
**6**	**RV4_01**	*Streptomyces scabiei*, NR_116531, (100)	9.287	43.194	-0.36	0.048
**7**	**RV4_19**	*Ohtaekwangia koreensis*, NR_117435, (95.3)	0.022	0.963	-0.67	0.000
**8**	**RV4_02**	*Steroidobacter denitrificans*, NR_044309, (94.9)	2.469	7.198	-0.35	0.055
**9**	**RV4_225**	*Emiticicia ginsengisoli*, NR_041373, (100)	0.189	0.003	0.61	0.000
**10**	**RV4_89**	*Acidibacter ferrireducens*, NR_126260, (94.9)	0.614	0.012	0.47	0.009
**11**	**RV4_05**	*Streptomyces bobili*, MT322150, (100)	7.759	0.017	0.49	0.006
**12**	**RV4_112**	*Asticcacaulis benevestitus*, NR_042433, (100)	0.364	0.016	0.56	0.001
**13**	**RV4_15**	*Actinoplans globisporus*, NR_112101, (99.6)	0.225	0.908	-0.31	0.098
**14**	**RV4_08**	*Thiohalobacter thiocyanaticus*, NR_116699, (94.9)	0.945	1.021	-0.09	0.654
**15**	**RV4_24**	*Niastella vici*, NR_148859, (98.8)	0.078	0.703	0.00	0.987
**16**	**RV4_99**	*Pelomonas saccharophila*, NR_114189, (100)	0.474	0.000	0.32	0.080
**17**	**RV4_07**	*Niastella gongjuensis*, NR_137250, (99.6)	0.666	1.460	-0.62	0.000
**18**	**ITS1_36**	*Cirrenalia_iberica*, NR_153487, (83)	0.000	0.866	0.03	0.881
**19**	**RV4_14**	*Hyphomicrobium zavarzinii*, NR_026429, (95.3)	0.404	1.024	-0.30	0.110
**20**	**RV4_215**	*Lacunisphaera anatis*, NR_146377, (99.2)	0.170	0.005	0.33	0.071
**21**	**RV4_123**	*Anaeromyxobacter dehalogenans*, NR_074927, (91.7)	0.241	0.024	0.39	0.033
**22**	**RV4_32**	*Steroidobacter denitrificans*, NR_044309, (98.0)	0.028	0.613	-0.44	0.015
**23**	**RV4_96**	*Rhizobacter sp*., MK067234, (100)	0.650	0.000	0.55	0.002
**24**	**RV4_238**	*Marinoscillum furvescens*, NR_113833, (95.3)	0.122	0.013	0.47	0.009
**25**	**RV4_22**	*Hydrogenophaga taeniospiralis*, NR_114131, (100)	0.000	0.804	-0.40	0.029
**26**	**RV4_18**	*Novosphingobium endophyticum*, NR_145844, (99.2)	0.401	0.886	-0.36	0.052
**27**	**RV4_52**	*Polaromonas eurypsychrophila*, NR_149767, (99.9)	0.000	0.345	-0.48	0.007
**28**	**RV4_137**	*Crocinitomix algicola*, NR_158097, (94.1)	0.014	0.098	-0.39	0.034
**29**	**RV4_68**	*Oryzisolibacter propanilivorax*, NR_158123, (99.2)	0.758	0.023	0.36	0.051
**30**	**ITS1_29**	*Cirrenalia_iberica*, NR_153487, (81)	0.115	0.473	0.16	0.394

^a^Identified among PRD-inducing and non-inducing soils. CSS normalized, filtered, and square-root-transformed microbial features of bacterial V4, and log-transformed fungal ITS1, and oomycete ITS2 ASVs were included in the RF model. Relative abundances were calculated separately for rarified bacterial V4, fungal ITS1, and oomycete ITS amplicon sets from non-treated soils.

## Discussion

This study used a four-stage approach to examine PRD etiology: (1) a greenhouse bioassay employing control, fumigation, and pasteurization treatments was used to determine whether each soil induced PRD in peach seedlings; (2) rRNA gene amplification and sequencing were used to examine the resulting peach root microbiomes associated with the seedlings; (3) the root-associated communities were contrasted to relate their composition to the preplant soil treatments and PRD-inducing capacities of the soils; and (4) a random forest machine learning approach was used to identify top microbial features that correctly discriminated between PRD-affected and non-affected soils. Dual pairs of rRNA gene primers were used to target bacteria, fungi, and oomycetes. These primer pairs generally provided similar views of the root microbiomes, strengthening confidence in the assessments. The inclusion of root systems from 10 different soils, and the use of two key sets of root community comparisons (i.e., one set involving roots from control vs. preplant pasteurized or fumigated soil, and the other set involving roots from PRD-inducing vs. non-inducing control soils) were considered to be strengths of this examination, increasing the likelihood that results of the comparisons may relate well to multiple replant soils.

Both sets of community comparisons offered insights into PRD etiology in California soils. In the first set, comparing root communities from fumigation and pasteurization treatments to those from untreated controls increased the likelihood that the libraries represented phenomena occurring in replanted orchards, where preplant fumigation is often practiced. Interestingly, pasteurization and fumigation treatments had similar effects on peach growth and root communities: both treatments revealed PRD induction in the same six of eight Prunus replant soils, a lack of PRD in two Prunus replant soils, and lack of PRD in the two grape replant soils. Such plant responses were not surprising in the bioassay, because under field conditions PRD is not induced in grape soils, and not all Prunus soils induce PRD (Browne, [unpublished]; [49]). Both fumigation and pasteurization resulted in clear shifts in root abundance of: Actinobacteria (reduced by both treatments, compared to control) and Gammaproteobacteria (increased by both treatments); Sordariomycetes (increased by both treatments) and the Pezizomycetes (decreased by both treatments); and oomycetes (decreased by both treatments). Both treatments reduced taxa richness and significantly impacted the composition of bacterial and fungal communities associated with peach roots, which agreed with previous reports of pronounced reductions in bacterial and fungal richness after certain preplant soil treatments [50–52]. Overall, the mitigation of peach seedling growth reduction by preplant fumigation and pasteurization, the significant changes in bacterial and fungal root community compositions as a result of these treatments, and the apparent scarcity of oomycetes in roots in soil of the fumigated and pasteurized soil samples strengthen evidence that PRD is mediated in part through microbial communities in host roots. Also, since the greenhouse bioassay used regular watering with nutrient solution, the greenhouse results suggested that the mediation of microbial communities may be important even when inorganic nutrients are provided amply by fertilization.

The second set of comparisons, which focused only on root communities from the non-treated soil samples, revealed that some of the general microbial root community shifts that occurred in response to preplant soil fumigation and pasteurization also were evident among roots from non-treated soils that failed to induce PRD, compared roots from non-treated soils that did induce PRD. For instance, roots from non-treated non-inducing soils had lower abundance of Actinobacteria and higher abundance of Gammaproteobacteria combined with lower abundance of Pezizomycetes and higher abundance of Sordariomycetes, all compared to roots from PRD-inducing soils—the same general microbial community trends that were observed in response to fumigation and pasteurization, compared to roots from the control treatment. Whereas oomycete abundances were too low to evaluate in the pasteurized and fumigated treatments of the first set of comparisons, the second set of comparisons offered an advantage that oomycete abundances could be included in RF modeling. Restricting the second set of comparisons to roots from non-treated soils avoided the relatively overwhelming effects of fumigation and pasteurization while still including PRD-affected and healthy plants.

We do not have root microbiome data that excludes nematode species from consideration in PRD induction. Nevertheless, in our previous report that related soil factors to PRD induction [19], we characterized the populations of nematodes in all 10 of the soils used in root microbiome examinations reported here and in 15 additional soils that were only part of the soil study; there was no relation between soil populations of nematode species and induction of PRD. Though our soil data suggest it unlikely, it is possible that adding root and soil nematode assays over the full course of the bioassay procedure that we used could reveal PRD contributions of nematode populations not apparent in this report.

A general conclusion suggested by the results of this study is that soil treatments such as fumigation and pasteurization overcome PRD by fostering broad shifts in peach root communities that, on balance, reduce the impacts of inhibitory or mildly parasitic root organisms and increase the impacts of stimulatory or protective root organisms. At a more specific level, our comparisons of root communities from non-treated PRD-inducing vs. non-inducing soils suggested particular ASVs of interest, either for a potential role in causing PRD or in mediating effects of the disease. These ASVs were identified based on their importance as RF predictors, high abundances in association with root systems from PRD-inducing or non-inducing soils, and negative correlations (PRD inducing) or positive correlations (non-inducing) between abundance and control top weight proportions.

Bacteria were the most represented microbial domain among the top 30 RF predictors in peach root communities. Among them, only ASVs of *Streptomyces scabiei* RV4_01, *Steroidobacter denitrificans* RV4_02, and *Streptomyces bobili* RV4_05 were observed with relative abundances >5% in PRD-affected or healthy roots. Our results suggest that the ASV of *Str*. *scabiei* RV4_01, with the highest relative abundance (mean of 40% in PRD-affected root systems), and the two ASVs of *S*. *denitrificans*, RV4_02 and RV4_32, should be explored via culturing and pathogenicity testing for potential contributions to PRD (S3 Fig). Conversely, the results suggest that *Str*. *bobili* RV4_05, which exhibited abundances positively correlated with control proportions of top weight, may worthy of testing for growth stimulation of peach seedlings in replant soils (S2 Fig).

Previous reports have highlighted the potential or known importance of Actinobacteria in the health of plants, including impacts on replant diseases of *Prunus*, apple, and rose [17,18,53]. In another amplicon-based inquiry involving PRD in one soil, Yang et al. [17] detected seven Actinobacteria that were negatively associated with peach shoot weights, but none of them was identified as a *Streptomyces* sp., and their relative abundances were all <0.001%. Interestingly, based on microscopic examinations nearly 40 years ago, actinomycetes were connected with ARD [54,55]. Also, recent amplicon-based examinations from controlled studies with ARD have highlighted the potential importance of several species of *Streptomyces* [18]. *Str*. *scabiei* (also known as *Str*. *scabies*) is a well-known cause of scab disease in vegetables (i.e., potato, beet, carrot, parsnip, radish, rutabaga, and turnip) and can inhibit growth of monocot and dicot seedlings [56–59]. *Streptomyces bobili* was reported to exhibit antimicrobial activity [60] and suppressed root rot in date palm trees [61], suggesting there may be merit for investigating it as a biocontrol agent on *Prunus* root systems. Many species of *Streptomyces* have been used as biocontrol agents and/or show potential for improving agricultural practice [62,63].

To our knowledge, none of the non-actinomycete bacterial ASVs that were identified as top RF predictors and had abundances significantly correlated with control top weight proportion are known to cause or suppress disease in plants. Of the predictive non-actinomycetes whose abundances were negatively correlated with control proportions of top weight (i.e., which would tend to indicate PRD induction) several ASVs had been reported from soil or roots [64–67], but none have been reported as causing plant disease. *Steroidobacter* species, including *S*. *denitrificans*, were described recently and are apparently common in some soils, but little is known about their potential impacts on plant health [66,67]. Similarly, except for *Rhizobium sophorae* RS4_030, little of agricultural significance has been reported among the other top RF non-actinomycete bacterial predictors whose abundances were positively correlated with control proportion of top weight. *Rhizobium sophorae* was reported as a nitrogen-fixing symbiont of a medicinal legume [68] but has not been reported from peach. To our knowledge, none of the top-30 RF predictive ASVs has been reported to mediate production of hydrogen cyanide, a feature highlighted in previous PRD studies.

Despite the prevalence of bacterial ASVs among informative RF predictors, two oomycete ASVs, *P*. *mamillatum* OOM2_12 and *P*. *mamillatum* OOM2_14, also stood out. In roots from non-treated soils that failed to induce PRD, their mean abundances of 5.5 and 1.5%, respectively (Table 5); whereas in roots from non-treated soils that did induce PRD, both of these ASVs were absent. Although certain other species of *Pythium* are known to suppress root diseases [69], we found no reports that *P*. *mamillatum* does so. In fact, *Pythium mamillatum* was reported as a “damping off” pathogen [70]. It is possible that *P*. *mamillatum* was suppressive of PRD or that it was merely better adapted to the environment of healthy peach roots than that of PRD-affected roots. It was surprising that our RF modeling did not reveal importance of additional oomycete ASVs, given their prominence in previous reports [14,16,17]; possible explanations for the different results may include soil and methodology differences among studies. For example, in the former study [17], oomycetes of interest were first cultured from root tips, then their quantities were examined using quantitative PCR.

Only two fungal ASVs, *Cirrenalia iberica* ITS1_36 and ITS1_29, were identified as top RF predictors. They were enriched in roots of PRD-affected plants (Table 5), but their abundances did not correlate significantly with control top weight proportions. This lack of prevalence of fungi among PRD predictors would not be expected based on previous reports, which associated several fungi with PRD incidence [15,17]. Again, differing soils and methods used may explain such differences in results and serve to emphasize the importance of complementation among different studies.

## Conclusions

A greenhouse bioassay provided a useful foundation for examining relationships between peach root communities and incidence of PRD. RF classification modeling based on abundances of ASVs resolved from rRNA gene amplicons enabled relatively precise discrimination between the microbial communities from roots affected by PRD and those that were not affected by the disease. Top RF-informative ASVs were mostly bacteria, but limited numbers of fungi and oomycetes also were informative in classification. Due to relatively high root abundances in association with PRD incidence, both *Streptomyces scabiei* and *Steroidobacter denitrificans* are of special interest for potential PRD induction roles, while due to high abundances in the absence of PRD, both *Streptomyces bobili* and *Pythium mamillatum* are of interest for potential roles in PRD suppression.

## Supporting information

S1 Fig Ordination of bacterial, fungal, and oomycete community samples from peach roots grown in six PRD-inducing and four non-inducing replant soils. Non-metric multidimensional scaling (NMDS) was used to ordinate the samples according to their ASV-level Bray-Curtis dissimilarities based on: **A**, V4 rRNA gene amplicons from bacteria (see materials and methods for detail), **B**, V5-V7 amplicons from bacteria; **C**, ITS1 amplicons from fungi, **D**, ITS2 amplicons from fungi; **E**, ITS1 amplicons from oomycetes; and **F**, ITS2 amplicons from oomycetes. The matrices were generated after cumulative sum scaling (CSS) normalization and square-root (bacterial amplicons) or natural log transformation (fungal and oomycete amplicons).
(TIF)

S2 Fig Boxplots depicting relative abundances of ASVs that were among the top-30 informative predictors identified by random forest classification modeling of root communities from non-treated (control) samples and had abundances positively correlated with control top weight proportion (r = 0.39 to 0.70, *P* <0.0001 to 0.03, Table 5). Relative abundances of **A**, the oomycete ASV, and **B-K**, the bacterial ASVs, were determined from their respective rarified amplicon sets (oomycete ITS2 and bacterial V4) and plotted separately for roots from non-inducing soils (green bars) and PRD inducing soils (orange bars).
(TIF)

S3 Fig Boxplots depicting relative abundances of ASVs that were among the top-30 informative predictors identified by random forest classification modeling of root communities from non-treated (control) samples and had abundances negatively correlated with control top weight proportion (r = -0.39 to -0.67, *P* <0.0001 to 0.05, Table 5). Relative abundances of **A-I**, bacterial ASVs and **J**, the fungal ASV, were determined from their respective rarified amplicons sets (V4 and ITS1) and plotted separately for roots from non-inducing (green bars) and PRD-inducing soils (orange bars). Taxa names are based on NCBI Blast hits and assigned ASVs labels.
(TIF)

S4 Fig Boxplots depicting relative abundances of ASVs that were RF-model informative and had abundances in control soil roots that correlated negatively correlated with control top weight proportions. The abundances, shown as a function of preplant soil treatment, represent ASVs from rarified V4 amplicon sets.
(TIF)

S5 Fig Boxplots depicting relative abundances of ASVs that were RF-model informative and had abundances in control soil roots that positively correlated with top control top weight proportions. The abundances, shown as a function of preplant soil treatment, represent ASVs from rarified V4 amplicon sets.
(TIF)

S1 Table Soils used in the greenhouse bioassay for Prunus replant disease and associated root microbial community characterizations. (DOCX)

S2 Table Primers used to amplify marker genes of bacteria, fungi and oomycetes from root communities. (DOCX)

S3 Table Root community representations, showing read counts for each primer set in all treatments. (DOCX)

## References

[1] Westerdahl BB, McKenry MV. Diseases caused by nematodes. In: Teviotdale BL, Michailides TJ, Pscheidt JW, editors. Compendium of nut crop diseases in temperate zones. St. Paul: APS Press; 2002. p. 11–4.

[2] McKenry MV, Kretsch J. Survey of nematodes associated with almond production in California. Plant Disease. 1987;71(1):71–3.

[3] McKenry MV. The replant problem and its management. Fresno, CA: Catalina Publishing; 1999. 124 p.

[4] Ogawa JM, English H. Diseases of temperate zone tree fruit and nut crops. Oakland: Univ. California Div. Agric. Natural Resources Publication; 1991. 3345 p.

[5] Browne GT, Lampinen BD, Holtz BA, Doll DA, Upadhyaya SK, Schmidt LS, et al. Managing the almond and stone fruit replant disease complex with less soil fumigant. California Agriculture. 2013; 67((3)):128–38.

[6] Browne GT, Connell JH, Schneider SM. Almond replant disease and its management with alternative pre-plant soil fumigation treatments and rootstocks. Plant Disease. 2006;90:869–76. doi: 10.1094/PD-90-0869 30781023

[7] Browne G, Ott N, Poret-Peterson A, Gouran H, Lampinen B. Efficacy of Anaerobic Soil Disinfestation for Control of Prunus Replant Disease. Plant Disease. 2017;102(1):209–19. doi: 10.1094/PDIS-09-16-1392-RE 30673462

[8] Gur A, Cohen, Y. The peach replant problem- some causal agents. Soil Biology and Biochemistry. 1989;21:829–34.

[9] Patrick ZA. The peach replant problem in Ontario. II. Toxic substances from the microbial decomposition products of peach root residues. Canadian Journal of Botany. 1955;33:461–86.

[10] Zhu W, Liu J, Ye J, Li G. Effects of phytotoxic extracts from peach root bark and benzoic acid on peach seedlings growth, photosynthesis, antioxidance and ultrastructure properties. Scientia Horticulturae. 2017;215:49–58. 10.1016/j.scienta.2016.12.004.

[11] Mizutani F, Hirota R, Kadoya K. Growth inhibiting substanes from peach roots and their possible involvement in peach replant problems. Acta Horticulturae. 1988;233:37–43.

[12] Benizri E, Piutti S, Verger S, Pages L, Vercambre G, Poessel JL, et al. Replant diseases: Bacterial community structure and diversity in peach rhizosphere as determined by metabolic and genetic fingerprinting. Soil Biology and Biochemistry. 2005;37:1738–46.

[13] Hine RB. The role of amygdalin breakdown in the peach replant problem. Phytopathology. 1961;51:10–3.

[14] Hine RB. The role of fungi in the peach replant problem. Plant Disease Reporter. 1961;45:462–6.

[15] Wensley RN. The peach replant problem in Ontario IV. Fungi associated with replant failure and their importance in fumigated and non-fumigated soils. Canadian Journal of Botany. 1956;34:967–81.

[16] Bent E, Loffredo A, Yang JH, McKenry MV, Becker JO, Borneman J. Investigations into peach seedling stunting caused by a replant soil. FEMS Microbiology Ecology. 2009;68:192–200. doi: 10.1111/j.1574-6941.2009.00668.x 19573200

[17] Yang J-i Ruegger PM, McKenry MV Becker JO, Borneman J. Correlations between Root-Associated Microorganisms and Peach Replant Disease Symptoms in a California Soil. Plos One. 2012;7(10):e46420. doi: 10.1371/journal.pone.0046420 23071565 PMC3465339

[18] Mahnkopp-Dirks F, Radl V, Kublik S, Gschwendtner S, Schloter M, Winkelmann T. Molecular Barcoding Reveals the Genus Streptomyces as Associated Root Endophytes of Apple (Malus domestica) Plants Grown in Soils Affected by Apple Replant Disease. Phytobiomes Journal. 2021;5(2):177–89. doi: 10.1094/pbiomes-07-20-0053-r

[19] Khan AR, Wicaksono WA, Ott NJ, Poret-Peterson AT, Browne GT. Characterization of soils conducive and non-conducive to Prunus replant disease. PLOS ONE. 2021;16(12):e0260394. doi: 10.1371/journal.pone.0260394 34890412 PMC8664177

[20] Browne G, Ott N, Poret-Peterson A, Gouran H, Lampinen B. Efficacy of Anaerobic Soil Disinfestation for Control of Prunus Replant Disease. Plant Disease. 2018;102(1):209–19. doi: 10.1094/PDIS-09-16-1392-RE . 30673462

[21] Hoagland D. The water-culture method for growing plants without soil Berkeley, Calif.: University of California, College of Agriculture, Agricultural Experiment Station.; 1928.

[22] Caporaso JG, Lauber CL, Walters WA, Berg-Lyons D, Lozupone CA, Turnbaugh PJ, et al. Global patterns of 16S rRNA diversity at a depth of millions of sequences per sample. Proc Natl Acad Sci U S A. 2011;108 Suppl 1(Suppl 1):4516–22. Epub 2010/06/11. doi: 10.1073/pnas.1000080107 ; PubMed Central PMCID: PMC3063599. 20534432 PMC3063599

[23] Bodenhausen N, Horton MW, Bergelson J. Bacterial Communities Associated with the Leaves and the Roots of Arabidopsis thaliana. PLOS ONE. 2013;8(2):e56329. doi: 10.1371/journal.pone.0056329 23457551 PMC3574144

[24] Gardes M, Bruns TD. ITS primers with enhanced specificity for basidiomycetes—application to the identification of mycorrhizae and rusts. Mol Ecol. 1993;2(2):113–8. Epub 1993/04/01. doi: 10.1111/j.1365-294x.1993.tb00005.x . 8180733

[25] Riit T, Tedersoo L, Drenkhan R, Runno-Paurson E, Kokko H, Anslan S. Oomycete-specific ITS primers for identification and metabarcoding. MycoKeys. 2016;14:17–30. 10.3897/mycokeys.41.30558 PMC6232247 30431624

[26] Hamady M, Walker JJ, Harris JK, Gold NJ, Knight R. Error-correcting barcoded primers for pyrosequencing hundreds of samples in multiplex. Nat Methods. 2008;5(3):235–7. Epub 2008/02/12. doi: 10.1038/nmeth.1184 ; PubMed Central PMCID: PMC3439997. 18264105 PMC3439997

[27] Gohl DM, Vangay P, Garbe J, MacLean A, Hauge A, Becker A, et al. Systematic improvement of amplicon marker gene methods for increased accuracy in microbiome studies. Nat Biotechnol. 2016;34(9):942–9. Epub 2016/07/28. doi: 10.1038/nbt.3601 . 27454739

[28] Ihrmark K, Bödeker IT, Cruz-Martinez K, Friberg H, Kubartova A, Schenck J, et al. New primers to amplify the fungal ITS2 region—evaluation by 454-sequencing of artificial and natural communities. FEMS Microbiol Ecol. 2012;82(3):666–77. Epub 2012/06/29. doi: 10.1111/j.1574-6941.2012.01437.x . 22738186

[29] Schneider CA, Rasband WS, Eliceiri KW. NIH Image to ImageJ: 25 years of image analysis. Nature methods 2012;9 ((7)):671–5. doi: 10.1038/nmeth.2089 22930834 PMC5554542

[30] Bokulich NA, Mills DA. Improved Selection of Internal Transcribed Spacer-Specific Primers Enables Quantitative, Ultra-High-Throughput Profiling of Fungal Communities. Applied and Environmental Microbiology. 2013;79(8):2519–26. doi: 10.1128/AEM.03870-12 23377949 PMC3623200

[31] Bolyen E, Rideout JR, Dillon MR, Bokulich NA, Abnet CC, Al-Ghalith GA, et al. Reproducible, interactive, scalable and extensible microbiome data science using QIIME 2. Nature Biotechnology. 2019;37(8):852–7. doi: 10.1038/s41587-019-0209-9 31341288 PMC7015180

[32] Martin M. Cutadapt removes adapter sequences from high-throughput sequencing reads. 2011. 2011;17(1):3. Epub 2011-08-02. doi: 10.14806/ej.17.1.200

[33] Callahan BJ, McMurdie PJ, Rosen MJ, Han AW, Johnson AJA, Holmes SP. DADA2: High-resolution sample inference from Illumina amplicon data. Nature Methods. 2016;13:581. doi: 10.1038/nmeth.3869 https://www.nature.com/articles/nmeth.3869#supplementary-information. 27214047 PMC4927377

[34] Wang Q, Garrity GM, Tiedje JM, Cole JR. Naive Bayesian classifier for rapid assignment of rRNA sequences into the new bacterial taxonomy. Appl Environ Microbiol. 2007;73(16):5261–7. Epub 2007/06/26. doi: 10.1128/AEM.00062-07 ; PubMed Central PMCID: PMC1950982. 17586664 PMC1950982

[35] Pruesse E, Quast C, Knittel K, Fuchs BM, Ludwig W, Peplies J, et al. SILVA: a comprehensive online resource for quality checked and aligned ribosomal RNA sequence data compatible with ARB. Nucleic acids research. 2007;35(21):7188–96. Epub 2007/10/18. doi: 10.1093/nar/gkm864 . 17947321 PMC2175337

[36] Kõljalg U, Nilsson RH, Abarenkov K, Tedersoo L, Taylor AF, Bahram M, et al. Towards a unified paradigm for sequence-based identification of fungi. Mol Ecol. 2013;22(21):5271–7. Epub 2013/10/12. doi: 10.1111/mec.12481 . 24112409

[37] Altschul SF, Gish W, Miller W, Myers EW, Lipman DJ. Basic local alignment search tool. Journal of Molecular Biology. 1990;215(3):403–10. doi: 10.1016/S0022-2836(05)80360-2 2231712

[38] Team RC. R: A Language and Environment for Statistical Computing. R Foundation for Statistical Computing, Vienna, Austria.2019.

[39] Team R. RStudio: Integrated Development for R. RStudio, PBC, Boston, MA; 2021.

[40] Clarke K, Gorley R. PRIMER version 7: User Manual/Tutorial. *PRIMER-e*, Devon, UK 2015.

[41] Hammer O, Harper DAT, Ryan PD. PAST: Paleontological statistics software package for education and data analysis. Palaeontol Electron. 2001;4(9).

[42] McMurdie PJ, Holmes S. phyloseq: an R package for reproducible interactive analysis and graphics of microbiome census data. PLoS One. 2013;8(4):e61217. Epub 2013/05/01. doi: 10.1371/journal.pone.0061217 ; PubMed Central PMCID: PMC3632530. 23630581 PMC3632530

[43] Oksanen J, Blanchet F, Kindt R, Legendre P, RVP O’Hara-. Vegan: Community ecology package. R Packag. 2.3–3. Available at: https://CRANR-projectorg/package=vegan. 2016. doi: 10.4135/9781412971874.n145

[44] Paulson JN, Stine OC, Bravo HC, Pop M. Differential abundance analysis for microbial marker-gene surveys. Nature Methods. 2013;10(12):1200–2. doi: 10.1038/nmeth.2658 24076764 PMC4010126

[45] Paulson JN, Talukder H, Corrada Bravo H. Longitudinal differential abundance analysis of microbial marker-gene surveys using smoothing splines. bioRxiv. 2017:099457. doi: 10.1101/099457

[46] Breiman L. Random Forests. Machine Learning. 2001;45(1):5–32. doi: 10.1023/A:1010933404324

[47] Liaw A, Wiener M, editors. Classification and Regression by randomForest 2007.

[48] Douglas G. Random Forests Tutorial. 2017.

[49] Khan AR, Wicaksono WA, Ott NJ, Poret-Peterson AT, Browne GT. Characterization of soils conducive and non-conducive to Prunus replant disease Plos One. 202x;X:x–xx. 10.1371/journal.pone.0260394 PMC8664177 34890412

[50] Chen L, Luo Y, Xu J, Yu Z, Zhang K, Brookes PC. Assessment of Bacterial Communities and Predictive Functional Profiling in Soils Subjected to Short-Term Fumigation-Incubation. Microb Ecol. 2016;72(1):240–51. Epub 2016/04/16. doi: 10.1007/s00248-016-0766-0 . 27079454

[51] Wei F, Passey T, Xu X. Amplicon-based metabarcoding reveals temporal response of soil microbial community to fumigation-derived products. Applied Soil Ecology. 2016;103:83–92. 10.1016/j.apsoil.2016.03.009.

[52] Shennan C, Muramoto J, Koike S, Baird G, Fennimore S, Samtani J, et al. Anaerobic soil disinfestation is an alternative to soil fumigation for control of some soilborne pathogens in strawberry production. Plant Pathology. 2018;67(1):51–66. 10.1111/ppa.12721.

[53] Yim B, Baumann A, Grunewaldt-Stöcker G, Liu B, Beerhues L, Zühlke S, et al. Rhizosphere microbial communities associated to rose replant disease: links to plant growth and root metabolites. Horticulture Research. 2020;7(1):144. doi: 10.1038/s41438-020-00365-2 32922816 PMC7459328

[54] Westcott SW III, Beer SV, Stiles WC. Infection of apple roots by actinomycetes associated with soils conducive to apple replant disease. Plant Disease. 1986;70(12):1125–8. PubMed PMID: 871330909.

[55] Westcott SW III, Beer SV, Israel HW. Interactions between actinomycete-like organisms and young apple roots grown in soil conducive to apple replant disease. Phytopathology. 1987;77(7):1071–7. PubMed PMID: 881341711.

[56] Leiner RH, Fry BA, Carling DE, Loria R. Probable Involvement of Thaxtomin A in Pathogenicity of Streptomyces scabies on Seedlings. Phytopathology. 1996;86:709–13. doi: 10.1094/Phyto-86-709

[57] Goyer C, Beaulieu C. Host Range of Streptomycete Strains Causing Common Scab. Plant Dis. 1997;81(8):901–4. Epub 1997/08/01. doi: 10.1094/PDIS.1997.81.8.901 . 30866378

[58] Loria R, Bukhalid RA, Fry BA, King RR. PLANT PATHOGENICITY IN THE GENUS STREPTOMYCES. Plant Dis. 1997;81(8):836–46. Epub 1997/08/01. doi: 10.1094/PDIS.1997.81.8.836 . 30866367

[59] Lerat S SIMAO‐BEAUNOIR AM, Beaulieu C. Genetic and physiological determinants of Streptomyces scabies pathogenicity. Molecular plant pathology. 2009;10(5):579–85. doi: 10.1111/j.1364-3703.2009.00561.x 19694949 PMC6640508

[60] Shoukry AA, Khatab IA, Faried MA, Khattab AA. Isolation, Evaluation and Molecular Identification of Streptomyces Isolates with Antimicrobial Activities. Journal of Agricultural Chemistry and Biotechnology. 2019;10(5):103–12. doi: 10.21608/jacb.2019.42965

[61] Arafat KH, Mohamed AM, Elsharabasy S. Biological of date palm root rots disease using Egyptian isolates of streptomycetes. Research Journal of Agriculture and Biological Sciences. 2012;8(2):224–30.

[62] Vurukonda SSKP, Giovanardi D, Stefani E. Plant Growth Promoting and Biocontrol Activity of Streptomyces spp. as Endophytes. International Journal of Molecular Sciences. 2018;19(4):952. doi: 10.3390/ijms19040952 29565834 PMC5979581

[63] Olanrewaju OS, Babalola OO. Streptomyces: implications and interactions in plant growth promotion. Applied Microbiology and Biotechnology. 2019;103(3):1179–88. doi: 10.1007/s00253-018-09577-y 30594952 PMC6394478

[64] Weon H-Y, Kim B-Y, Yoo S-H, Lee S-Y, Kwon S-W, Go S-J, et al. Niastella koreensis gen. nov., sp. nov. and Niastella yeongjuensis sp. nov., novel members of the phylum Bacteroidetes, isolated from soil cultivated with Korean ginseng. International Journal of Systematic and Evolutionary Microbiology. 2006;56(8):1777–82. doi: 10.1099/ijs.0.64242-0 16902007

[65] Yoon J-H, Kang S-J, Lee S-Y, Lee J-S, Park S. Ohtaekwangia koreensis gen. nov., sp. nov. and Ohtaekwangia kribbensis sp. nov., isolated from marine sand, deep-branching members of the phylum Bacteroidetes. International Journal of Systematic and Evolutionary Microbiology. 2011;61(5):1066–72. doi: 10.1099/ijs.0.025874-0 20511453

[66] Sakai M, Hosoda A, Ogura K, Ikenaga M. The growth of Steroidobacter agariperforans sp. nov., a novel agar-degrading bacterium isolated from soil, is enhanced by the diffusible metabolites produced by bacteria belonging to Rhizobiales. Microbes and environments. 2014;29(1):89–95. Epub 2014/03/14. doi: 10.1264/jsme2.me13169 ; PubMed Central PMCID: PMC4041242. 24621511 PMC4041242

[67] Gong ZL, Zhang CF, Jin R, Zhang YQ. Steroidobacter flavus sp. nov., a microcystin-degrading Gammaproteobacterium isolated from soil. Antonie Van Leeuwenhoek. 2016;109(8):1073–9. Epub 2016/05/07. doi: 10.1007/s10482-016-0706-5 . 27151048

[68] Jiao YS, Yan H, Ji ZJ, Liu YH, Sui XH, Wang ET, et al. Rhizobium sophorae sp. nov. and Rhizobium sophoriradicis sp. nov., nitrogen-fixing rhizobial symbionts of the medicinal legume Sophora flavescens. International Journal of Systematic and Evolutionary Microbiology. 2015;65(Pt_2):497–503. doi: 10.1099/ijs.0.068916-0 25385989

[69] Gerbore J, Benhamou N, Vallance J, Le Floch G, Grizard D, Regnault-Roger C, et al. Biological control of plant pathogens: advantages and limitations seen through the case study of Pythium oligandrum. Environmental Science and Pollution Research. 2014;21(7):4847–60. doi: 10.1007/s11356-013-1807-6 23695856

[70] Paul B, Charles R, Bhatnagar T. Biological control of Pythium mamillatum causing damping-off of cucumber seedlings by a soil bacterium, Bacillus mycoides. Microbiological Research. 1995;150(1):71–5. 10.1016/S0944-5013(11)80036-4.

