# Supplementary figures and images for: Random forest analysis reveals taxa predictive of Prunus replant disease in peach root microbiomes
Source: PLoS One. 2022 Oct 13;17(10):e0275587. doi: 10.1371/journal.pone.0275587 (PMC9560047; doi:10.1371/journal.pone.0275587)

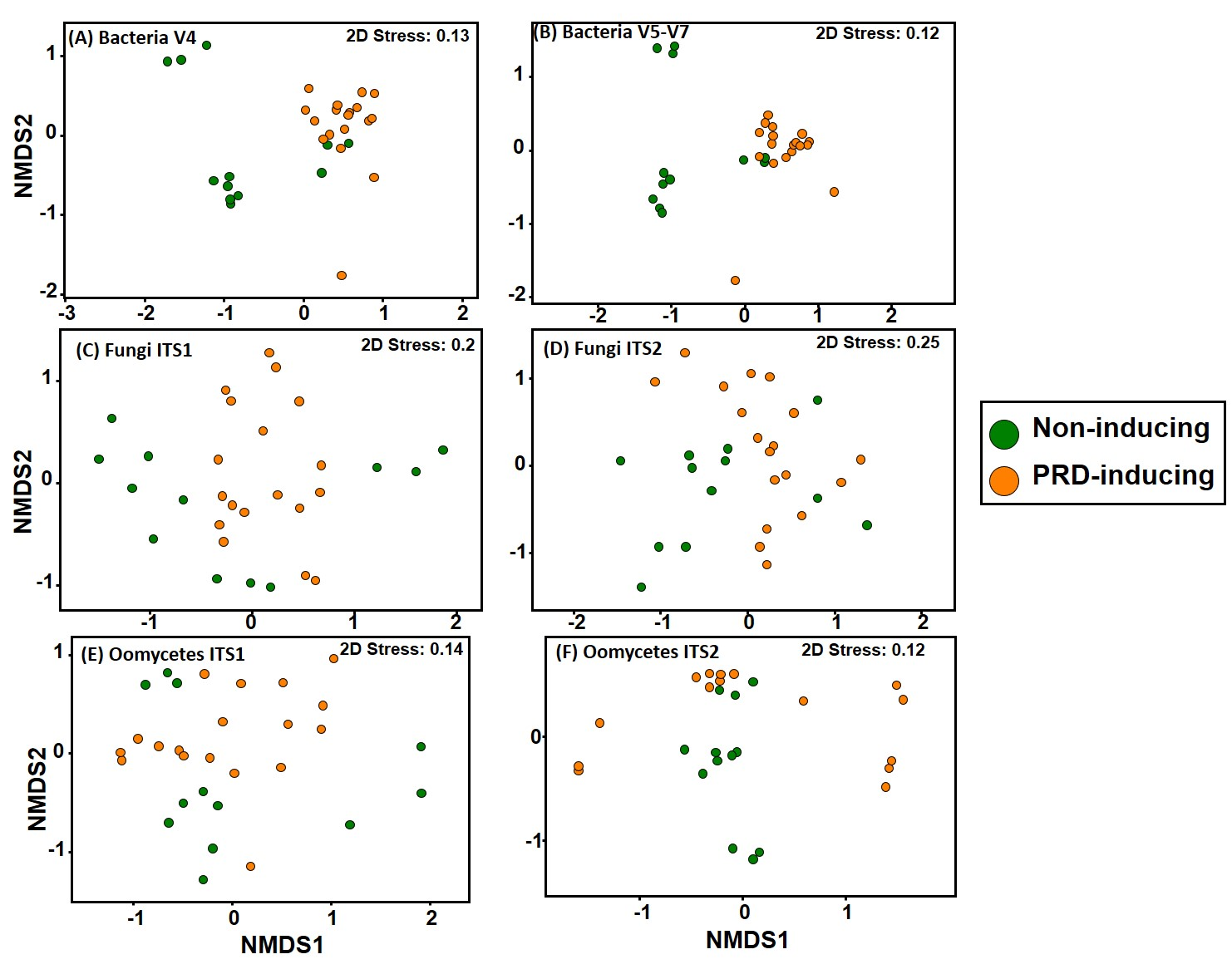

Supplement: S1 Fig — Non-metric multidimensional scaling (NMDS) was used to ordinate the samples according to their ASV-level Bray-Curtis dissimilarities based on: A, V4 rRNA gene amplicons from bacteria (see materials and methods for detail), B, V5-V7 amplicons from bacteria; C, ITS1 amplicons from fungi, D, ITS2 amplicons from fungi; E, ITS1 amplicons from oomycetes; and F, ITS2 amplicons from oomycetes. The matrices were generated after cumulative sum scaling (CSS) normalization and square-root (bacterial amplicons) or natural log transformation (fungal and oomycete amplicons). (TIF) [file pone.0275587.s001.tif]

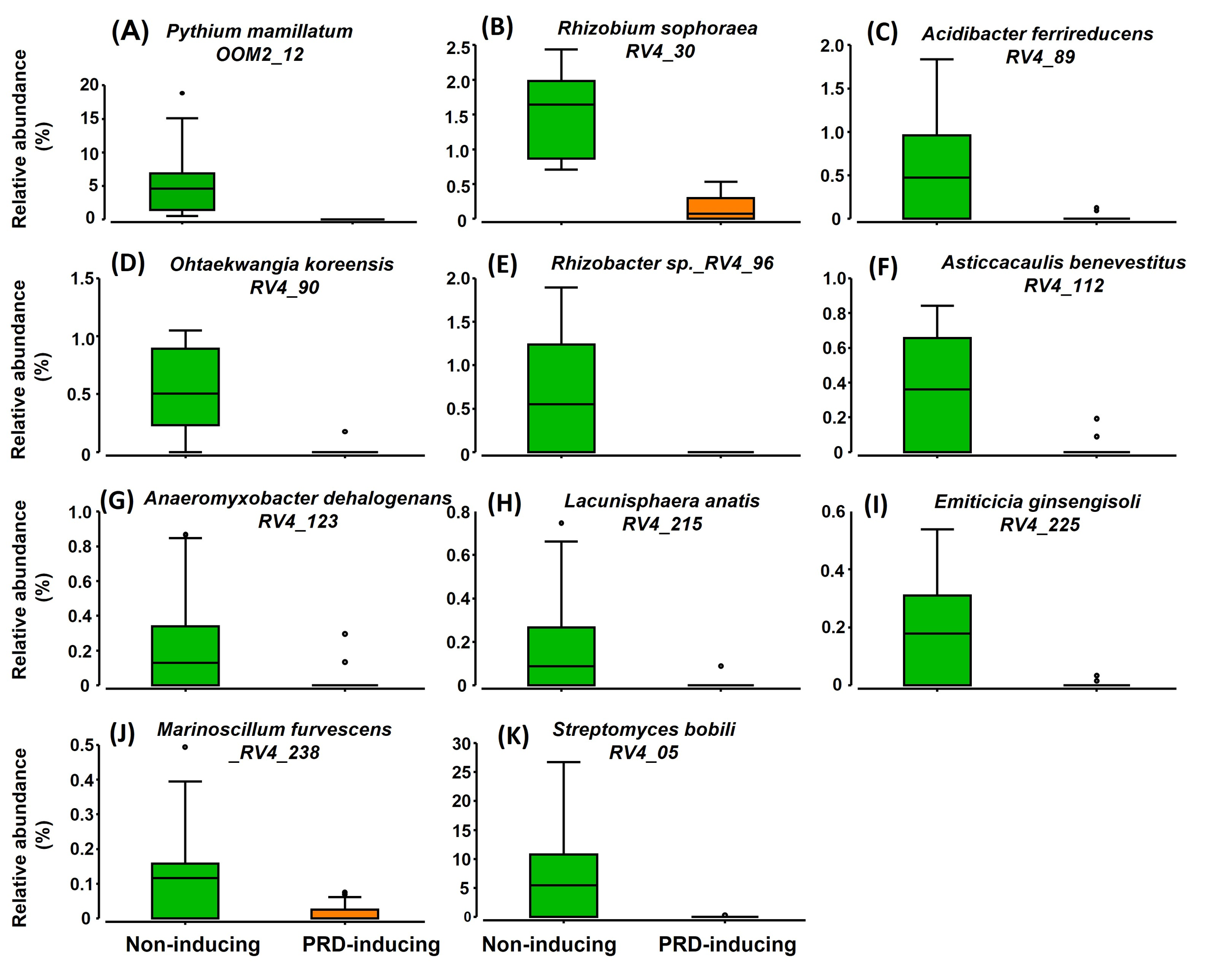

Supplement: S2 Fig — Relative abundances of A, the oomycete ASV, and B-K, the bacterial ASVs, were determined from their respective rarified amplicon sets (oomycete ITS2 and bacterial V4) and plotted separately for roots from non-inducing soils (green bars) and PRD inducing soils (orange bars). (TIF) [file pone.0275587.s002.tif]

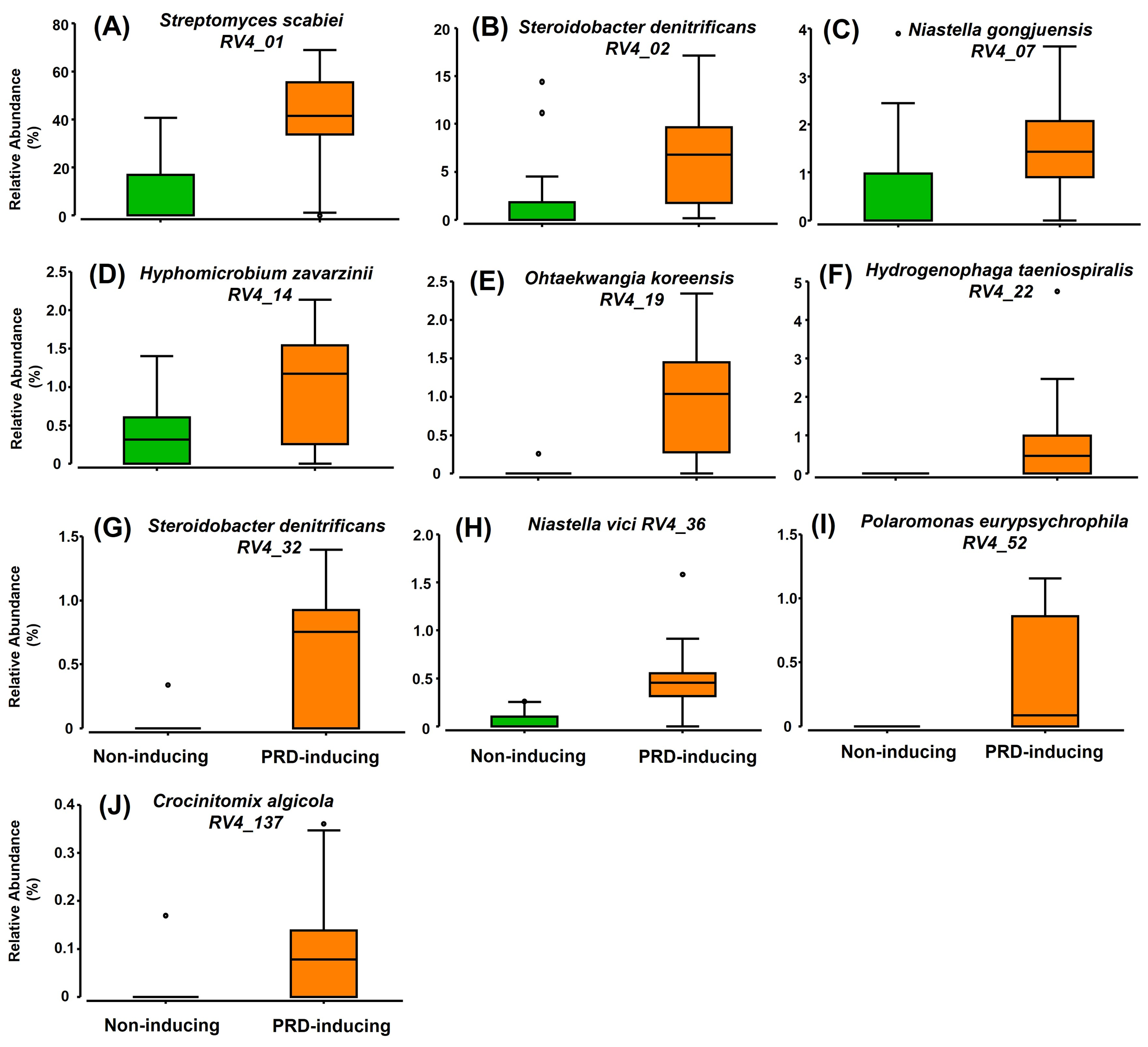

Supplement: S3 Fig — Relative abundances of A-I, bacterial ASVs and J, the fungal ASV, were determined from their respective rarified amplicons sets (V4 and ITS1) and plotted separately for roots from non-inducing (green bars) and PRD-inducing soils (orange bars). Taxa names are based on NCBI Blast hits and assigned ASVs labels. (TIF) [file pone.0275587.s003.tif]

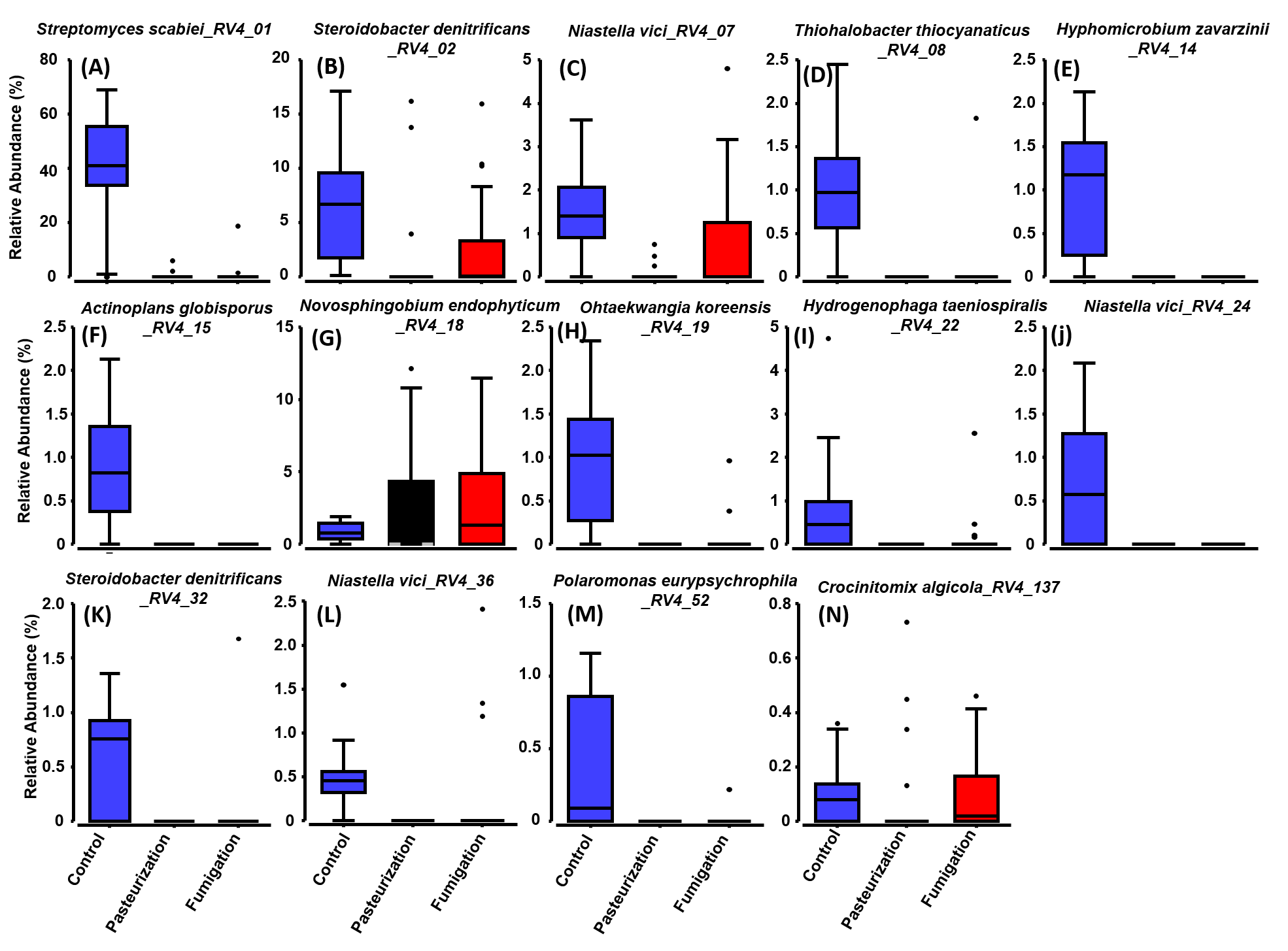

Supplement: S4 Fig — The abundances, shown as a function of preplant soil treatment, represent ASVs from rarified V4 amplicon sets. (TIF) [file pone.0275587.s004.tif]

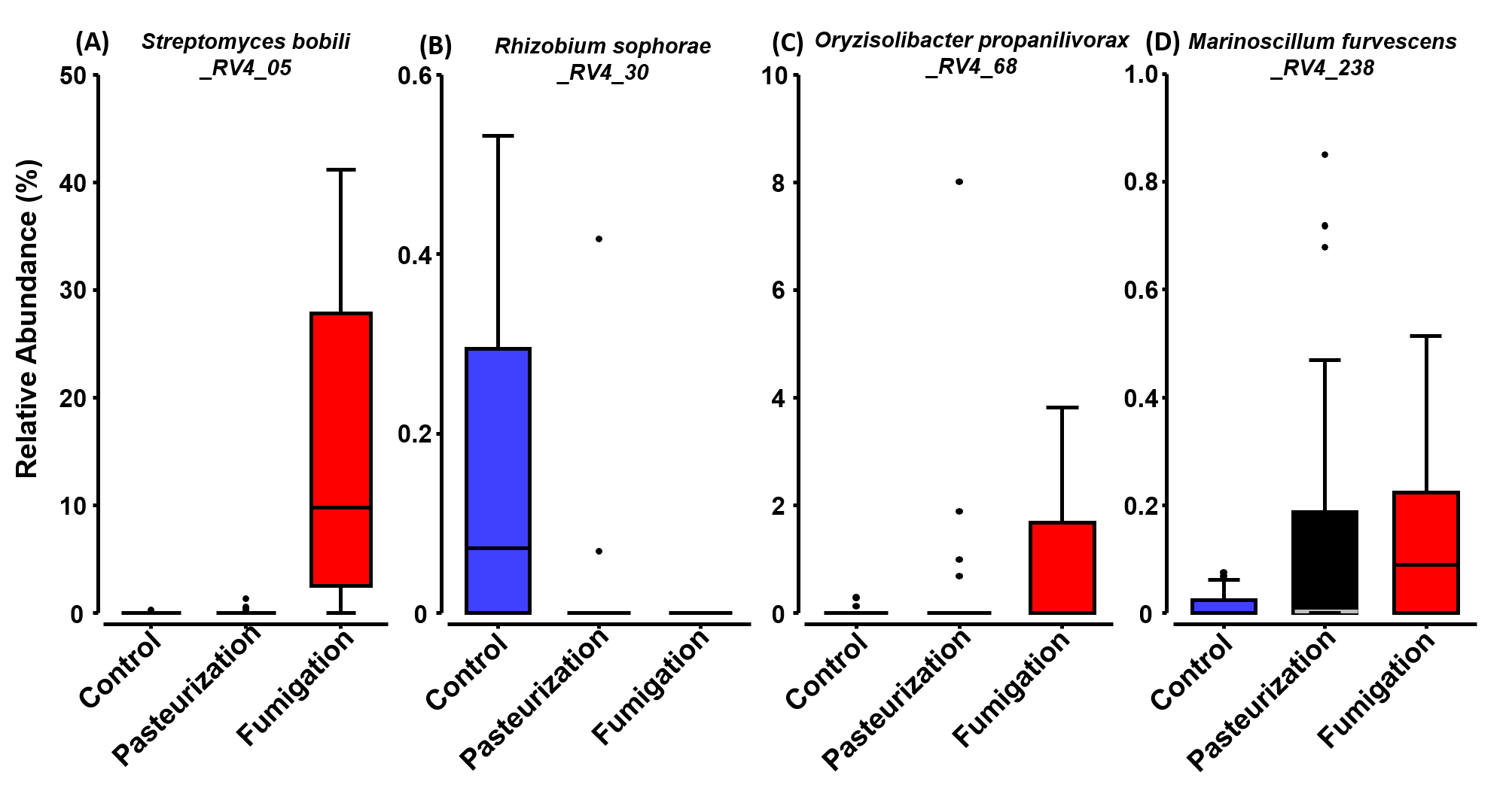

Supplement: S5 Fig — The abundances, shown as a function of preplant soil treatment, represent ASVs from rarified V4 amplicon sets. (TIF) [file pone.0275587.s005.tif]
