# Supplementary material for: Random forest analysis reveals taxa predictive of Prunus replant disease in peach root microbiomes
Source: PLoS One. 2022 Oct 13;17(10):e0275587. doi: 10.1371/journal.pone.0275587 (PMC9560047; doi:10.1371/journal.pone.0275587)
Supplement: S1 Table — (DOCX) [file pone.0275587.s006.docx]

**S1 Table.** Soils used in greenhouse bioassay for Prunus replant disease and associated root microbial community characterizations

| **Soil no.** | **County-site** | **GPS coordinates of field sampled** | **Crop history^a^** | **Soil texture classification** |
| --- | --- | --- | --- | --- |
| 1 | Butte-1 | 39°36'58.79" N 121°49'46.74" W | Al/Lov 11 yr | clay loam |
| 3 | Butte-3 | 39°36'29.27" N 121°48'22.71" W | Al/Lov >20 yr | sandy loam |
| 6 | Colusa-1 | 38°57'59.00" N 122°04'24.70" W | Al/Nem 6 yr | sandy loam |
| 10 | Fresno-1 | 36°51'00.35" N 120°19'37.48" W | Al/Nem 8 yr | sandy loam |
| 11 | Fresno-2 | 36°35'58.84" N 119°30'27.65" W | Vin >20 yr | sandy loam |
| 12 | Fresno-3 | 36°36'09.17" N 119°30'19.36" W | Vin >20 yr | sandy loam |
| 13 | Fresno-4A | 36°35'57.92"N 119°30'56.92"W | Pe/Nem 12 yr | sandy loam |
| 14 | Fresno-4B | 36°35'57.91"N 119°30'54.85"W | Pe/Nem 12 yr | sandy loam |
| 15 | Fresno-4C | 36°35'57.93"N 119°30'55.79"W | Pe/Nem 12 yr | sandy loam |
| 23 | Kern-2 | 35°30'21.43"N 119° 9'26.07"W | Al/Nem >20 yr | sandy loam |

^a^ In code, “Al/Lov” indicates orchard was almond trees on Lovell peach rootstock; “Al/Nem” indicates orchard was almond trees on Nemaguard peach rootstock’ “Vin” indicates grape vineyard; and “Pe/Nem” indicates orchard was peach trees on Nemaguard rootstock. Trees had been removed several months before collection of soils 13-15, whereas crops were still standing in all other sampled soils. Soils 13, 14, and 15 were removed from the same field, but were from areas that had received separate soil treatments (control, fumigation, and anaerobic soil disinfestation, respectively).
