# Supplementary material for: Random forest analysis reveals taxa predictive of Prunus replant disease in peach root microbiomes
Source: PLoS One. 2022 Oct 13;17(10):e0275587. doi: 10.1371/journal.pone.0275587 (PMC9560047; doi:10.1371/journal.pone.0275587)
Supplement: S2 Table — (DOCX) [file pone.0275587.s007.docx]

**Table S2**. Primers used to amplify marker gene fragments from bacterial, fungal and oomycete communities^a^

| **Primer name** | **Tag sequence** | **Spacer sequence** | **Linker sequence** | **Target-specific sequence** |
| --- | --- | --- | --- | --- |
| **515F** | TCGTCGGCAGCGTCAGATGTGTATAAGAGACAG | none or A or AT or ATC | GT | GTGYCAGCMGCCGCGGTAA |
| **806R** | GTCTCGTGGGCTCGGAGATGTGTATAAGAGACAG | none or A or AT or ATG | CC | GGACTACNVGGGTWTCTAAT |
| **799F** | TCGTCGGCAGCGTCAGATGTGTATAAGAGACAG | none or A or AT or ATC | GT | AACMGGATTAGATACCCKG |
| **1193R** | GTCTCGTGGGCTCGGAGATGTGTATAAGAGACAG | none or A or AT or ATC | GC | ACGTCATCCCCACCTTCC |
| **ITS1f** | TCGTCGGCAGCGTCAGATGTGTATAAGAGACAG | none or A or AT or ATC | GG | CTTGGTCATTTAGAGGAAGTAA |
| **ITS2** | GTCTCGTGGGCTCGGAGATGTGTATAAGAGACAG | none or A or AT or ATG | CG | GCTGCGTTCTTCATCGATGC |
| **fITS2** | TCGTCGGCAGCGTCAGATGTGTATAAGAGACAG | none or A or AT or ATC | GG | GTGARTCATCGAATCTTTG |
| **ITS4** | GTCTCGTGGGCTCGGAGATGTGTATAAGAGACAG | none or A or AT or ATG | CG | CCTCCGCTTATTGATATGC |
| **ITS1oo** | TCGTCGGCAGCGTCAGATGTGTATAAGAGACAG | none or A or AT or ATC | AA | GGAAGGATCATTACCACA |
| **ITS7** | GTCTCGTGGGCTCGGAGATGTGTATAAGAGACAG | none or A or AT or ATG | CG | AGCGTTCTTCATCGATGTGC |
| **ITS3oo** | TCGTCGGCAGCGTCAGATGTGTATAAGAGACAG | none or A or AT or ATC | AT | AGTATGYYTGTATCAGTG |

^a^Note: In the first PCR (step 1), target-specific primers with “tails” at their 3’ ends were used to amplify DNA fragments from each sample. The tails, which were used to facilitate addition of barcode and Illumina flow cell adapters in the second PCR (step 2), consisted of a tag sequence (priming site for the second PCR), a variable-length spacer (to increase sequence diveristy), and a 2bp linker (to separate the target specific primer from the rest of the tail). Each primer used for the second PCR consisted of the Illumina adapter sequence joined at its 3’ end to an 8bp Hamming error-correcting barcode; each amplicon library, consisting of DNA fragments from one root sample, had a unique pair of barcodes.
