# Supplementary material for: Random forest analysis reveals taxa predictive of Prunus replant disease in peach root microbiomes
Source: PLoS One. 2022 Oct 13;17(10):e0275587. doi: 10.1371/journal.pone.0275587 (PMC9560047; doi:10.1371/journal.pone.0275587)
Supplement: S3 Table — (DOCX) [file pone.0275587.s008.docx]

| **Sample set** | **Primer set** | **Total number of samples^a^** | **Number of reads** | | | | | **Samples excluded^c^** |
| --- | --- | --- | --- | --- | --- | --- | --- | --- |
|  |  |  | **Total** | **Maximum per sample** | **Minimum per sample** | **Average per sample** | **Rarefication^b^** |  |
| All soil treatments | V4 Bacteria | 90 | 581,274 | 25,055 | 262 | 6,459 | 1,365 | 6 |
|  | V5-V7 Bacteria | 90 | 390,940 | 29,778 | 85 | 4,344 | 793 | 6 |
|  | ITS1 Fungi | 90 | 2,634,302 | 143,167 | 1,589 | 29,270 | 1,589 | 0 |
|  | ITS2 Fungi | 90 | 810,636 | 68,597 | 305 | 9,007 | 918 | 4 |
| Only control treatment | V4 Bacteria | 30 | 298,971 | 25,055 | 1,526 | 9,967 | 1,526 | 0 |
|  | V5-V7 Bacteria | 30 | 166,116 | 29,474 | 978 | 5,537 | 978 | 0 |
|  | ITS1 Fungi | 30 | 1,185,993 | 143,167 | 3,165 | 39,533 | 3,165 | 0 |
|  | ITS2 Fungi | 30 | 448,699 | 68,597 | 355 | 14,957 | 1,524 | 1 |
|  | ITS1 Oomycetes | 30 | 485,230 | 140,164 | 564 | 16,174 | 564 | 0 |
|  | ITS2 Oomycetes | 30 | 999,902 | 232,295 | 349 | 37,033 | 644 | 1 |

**S3 Table.** Summary of generated reads, rarefication used, and samples excluded as a function of sample set and primers

^a^The totals of 90 root samples included 30 from control, 30 from pasteurized, and 30 from fumigated soil treatments, whereas the totals of 30 root samples included only the 30 from the control soil treatment.

^b^Each set of primers was rarefied at the reads number provided in the rarefication column.

^c^Number of samples excluded after rarefication.
